# Predicting dementia from primary care records: A systematic review and meta-analysis

**DOI:** 10.1371/journal.pone.0194735

**Published:** 2018-03-29

**Authors:** Elizabeth Ford, Nicholas Greenslade, Priya Paudyal, Stephen Bremner, Helen E. Smith, Sube Banerjee, Shanu Sadhwani, Philip Rooney, Seb Oliver, Jackie Cassell

**Affiliations:** 1 Department of Primary Care and Public Health, Brighton and Sussex Medical School, Brighton, United Kingdom; 2 Lee Kong Chian School of Medicine, Nanyang Technological University, Singapore, Singapore; 3 Centre for Dementia Studies, Brighton and Sussex Medical School, Brighton, United Kingdom; 4 Department of Physics and Astronomy, University of Sussex, Brighton United Kingdom; Istituto Di Ricerche Farmacologiche Mario Negri, ITALY

## Abstract

**Introduction:**

Possible dementia is usually identified in primary care by general practitioners (GPs) who refer to specialists for diagnosis. Only two-thirds of dementia cases are currently recorded in primary care, so increasing the proportion of cases diagnosed is a strategic priority for the UK and internationally. Variables in the primary care record may indicate risk of developing dementia, and could be combined in a predictive model to help find patients who are missing a diagnosis. We conducted a meta-analysis to identify clinical entities with potential for use in such a predictive model for dementia in primary care.

**Methods and findings:**

We conducted a systematic search in PubMed, Web of Science and primary care database bibliographies. We included cohort or case-control studies which used routinely collected primary care data, to measure the association between any clinical entity and dementia. Meta-analyses were performed to pool odds ratios. A sensitivity analysis assessed the impact of non-independence of cases between studies.

From a sift of 3836 papers, 20 studies, all European, were eligible for inclusion, comprising >1 million patients. 75 clinical entities were assessed as risk factors for all cause dementia, Alzheimer’s (AD) and Vascular dementia (VaD). Data included were unexpectedly heterogeneous, and assumptions were made about definitions of clinical entities and timing as these were not all well described. Meta-analysis showed that neuropsychiatric symptoms including depression, anxiety, and seizures, cognitive symptoms, and history of stroke, were positively associated with dementia. Cardiovascular risk factors such as hypertension, heart disease, dyslipidaemia and diabetes were positively associated with VaD and negatively with AD. Sensitivity analyses showed similar results.

**Conclusions:**

These findings are of potential value in guiding feature selection for a risk prediction tool for dementia in primary care. Limitations include findings being UK-focussed. Further predictive entities ascertainable from primary care data, such as changes in consulting patterns, were absent from the literature and should also be explored in future studies.

## Introduction

### Dementia as a public health concern

With an aging population, dementia is becoming an increasingly important health issue in the United Kingdom (UK) and across the world. In 2015 it was estimated that 46.8 million people worldwide were living with dementia, and this number is expected to increase to 74.7 million in 2030 and 131.5 million in 2050.[1] The impact of this disorder on patients, their carers, families, and society is profound.[2]

### Benefits of timely diagnosis

The World Alzheimer’s Report 2011 highlights the benefits of early diagnosis in allowing people with dementia and their families to make plans for the future, before their disease becomes too advanced.[3] Additional benefits include timely access to information, advice and support, and the person with dementia being able to express their wishes in a way which helps them to maximise their quality of life. Likewise current symptomatic treatments and future disease-modifying medications are likely to have most effect if prescribed early in the illness.[3] Earlier diagnosis is also likely to delay entry to care homes, thus reducing the costs to society of institutional care [4] and possibly also contributing to quality of life, given that most elderly people express a preference to stay in their own home as long as is practicable. However, at the current time, diagnosis often happens at a late stage in the illness, or following a crisis (e.g. after hospitalisation due to a fall) when opportunities for maximising quality of life have passed.[3, 5] Higher diagnosis rates, and diagnosis earlier in the course of the disease, are strategic aims for the UK government and National Health Service (NHS), as described in the National Dementia Strategy,[6] Prime Minister’s Dementia Challenge,[7] NHS England Dementia Identification Scheme,[8] and the GP Dementia Toolkit.[9]

### The role of UK general practice in diagnosis

Diagnosis and medical care for people with dementia in the UK starts with recognition of symptoms by patients themselves, their families, or general practitioners (GPs/family physicians). Following referral, dementia symptoms are then investigated by memory assessment services or other specialist teams, where a diagnosis of dementia may be made. However, there is a “diagnosis gap” in general practice with only about a half to two-thirds of patients having a recorded dementia diagnosis, compared to numbers expected from epidemiological studies.[10, 11]

### Dementia risk tools and prediction models

Risk scores and clinical prediction models may help clinicians to identify patients at risk of conditions such as dementia earlier than would be the case in routine clinical practice. This is a valuable strategy where early intervention may slow the progression of a disease. A number of risk prediction tools have been developed to stratify patients by risk of dementia [12, 13], drawing on demographic, health, lifestyle, functioning and cognitive factors, as well as blood based biomarkers, genetics and brain imaging. These tools have shown variable performance, with Areas Under the Receiver Operative Characteristic Curve (AUROC) ranging from 0.48 to 0.91 [14]. The majority of models have been built based on white volunteer samples that tend to have biases such as high education levels and better health than the general population, and very few models have been validated in a setting different from the one they were developed in. Models have also not utilised within-subject trajectories on predictor variables, instead mainly using cross-sectional variables [12]. The cost implications of these models also vary widely, as for example, brain MRI scans are much more expensive than administering a short cognitive paper-based test. The cost of any risk tool will determine recommendations about the use of the risk tool in clinical practice [12]. Risk scores which use self-report measures, or particularly, routinely collected health data, will have a cost advantage over those which require expensive tests which are outside the realm of usual clinical practice.

### General practice patient records as a source of data for a prediction model

In the UK, GPs see registered patients throughout their lives and record longitudinal data in electronic records on medical and family history and ongoing medical events. GP patient records could therefore be a key data source for the development of a dementia risk prediction tool. GPs record data using a hierarchical system of codes covering symptoms, tests, referrals, medical history and diagnoses, as well as a complete record of prescribing and free text notes.[15] These facets of recording map onto the predictive factors explored in the development of previous tools. GP patient records are also available to researchers, as anonymised samples curated in databases such as The Clinical Practice Research Datalink (CPRD)[16] and The Health Improvement Network (THIN);[17] covering more than 6 million current UK patients.

The use of risk prediction tools in routinely collected health data may enable the early identification of those with dementia and those at higher risk of developing dementia. It could also improve the quality of patient records, by identifying patients who miss codes for their condition, for whatever reason. Once identified, patients can be added to disease registers and provided with ongoing proactive care. In research studies using electronic health record data, clinical prediction models are analogous to case detection algorithms. Algorithms can help to improve either the sensitivity or specificity (or both) of case detection, compared to a single code.

A few studies have attempted to create clinical risk prediction models for dementia from general practice data.[18–20] Walters et al. created a clinical prediction model for dementia from primary care data which had good discrimination for 60–79 year olds but various thresholds for high risk resulted in either a low sensitivity or a low positive predictive value.[20] This model performed poorly in patients over 80 years old. This team used 14 clinical variables, but many more conditions and medications have been associated with dementia. A model developed by another research team achieved a sensitivity of 72% and a specificity of 83%, but the predictors used were not published.[19]

GP patient record databases contain high dimensional data which is sparsely populated. Choosing predictors for a risk tool must generally be done *a priori*. With a view to improving upon previous risk prediction tools utilising general practice data, the aim of this study was to inform feature selection for a future dementia prediction tool. We aimed to study the literature to find clinical entities in general practice patient records which have previously been found to be associated with dementia. We conducted a systematic literature review to identify papers examining risk factors for dementia using primary care data, and then conducted a meta-analysis of all explored risk factors, in order to identify which were significantly associated with dementia.

## Methods

We followed the Preferred Reporting Items for Systematic Reviews and Meta-Analyses (PRISMA) guidelines.[21] The study protocol is available at: http://sro.sussex.ac.uk/67005/.

### Search strategy

Three searches were carried out. In the first search, PubMed and Web of Science, were searched between 7^th^ Janurary 2016 and 13^th^ February 2016 by author NG using the following search string: ((((dementia[Title/Abstract]) OR Alzheimer*[Title/Abstract])) AND (((("primary care"[Title/Abstract]) OR "Primary Health Care"[Title/Abstract]) OR family pract*[Title/Abstract]) OR general pract*[Title/Abstract])) AND (((diagnos*[Title/Abstract]) OR onset[Title/Abstract]) OR predict*[Title/Abstract]).

Second, Online bibliographies of publications from CPRD and THIN during June 2016 by EF using keywords “Dementia” and “Alzheimer*”. No date ranges for publications were specified.

Third, an updated search was conducted by EF on 26^th^ September 2017. PubMed and Web of Science, were searched using the search string defined above.

After duplicates were discarded, the following inclusion criteria were then applied:

1. Studies using routinely collected primary care data extracted from existing databases.
2. Studies that looked at all cause dementia, Alzheimer’s or vascular dementia as their outcome.
3. Studies that had a cohort or case-control design in which exposures to risk factors in dementia cases could be compared to exposures to risk factors in a control group reflective of the general practice population.
4. Risk factors were measured before dementia diagnosis.
5. Studies published in English.

Studies were excluded if they focused on: management, screening tools (except those developed using routine primary care data), imaging or novel biomarkers for dementia; the ability of GPs to diagnose dementia; barriers to diagnosis of dementia; concordance with guidelines; patient/caregiver experience of diagnosis; prediction of prognosis, survival or institutionalization; cognitive decline; presenting clinical guidance or consensus; were review articles; or were published in languages other than English.

Using these criteria, the articles extracted from the literature search were screened based on their title, then abstract, then finally full text analysis, by two authors (NG and EF).

### Quality and Bias Assessment

Bias was assessed by author EF using the Risk of Bias Assessment Tool for Nonrandomized Studies (RoBANS).[22] Each study is assigned a category of low risk, high risk or unclear risk in 6 domains: selection of participants, confounding variables, measurement of exposures, blinding of outcome assessments, incomplete outcome data and selective outcome reporting.

Study quality was assessed by author EF using the Newcastle-Ottawa scale for the assessment of the quality of nonrandomized studies.[23] Each study is assessed under 3 domains: Selection of cases/controls (4 items), comparability of cases/controls (1 item) and measurement of exposure (3 items). For each item a study can receive 1 or 2 stars, giving a total out of 10 stars. There are different items for assessing case-control and cohort studies, but each scale totals to 10 stars.

To examine the risk of publication bias within analyses, we performed funnel plots for comparisons which had 10 or more studies. The Cochrane handbook states that “as a rule of thumb, tests for funnel plot asymmetry should be used only when there are at least 10 studies included in the meta-analysis, because when there are fewer studies the power of the tests is too low to distinguish chance from real asymmetry”.[24]

### Data extraction

The following meta-data from studies were extracted into a table in Excel: author names, date of publication, country of origin, name of primary care database, data extraction period, type of dementia, case definition of dementia, number of cases and controls, control matching criteria, conditions assessed, medications assessed, follow up time, mean age of patients, and percentage of female patients.

The following study data were extracted by risk factor: total number of dementia cases, number of cases exposed to risk factor, total number of controls, and number of controls exposed to risk factor. Firstly, all results in each study were combined for a category of “all dementia”. Data on Alzheimer’s disease (AD) and Vascular Dementia (VaD) were extracted separately where available. For medication data, the value for “ever prescribed” was extracted from all studies, as dosage format varied. For eight studies where study data were not published in an extractable format, we emailed authors, resulting in data on three studies.

### Meta-analysis

ReviewManager 5.3 was used to perform meta-analyses and estimate odds ratios (ORs) for all risk factors. Initially a fixed effects analysis was conducted for each meta-analysis, but where results were heterogeneous (I^2^ > 50%) a random effects analysis was used and is presented. The I^2^ statistic describes the percentage of variation across studies that is due to heterogeneity rather than chance.[24] No correction for multiple comparisons was performed as the outcome were analysed as stated in the study protocol and no post hoc analysis was performed.

### Sensitivity analysis

Twelve papers included in the analysis drew their data from the same database (CPRD). Resampling may artificially increase the precision of estimates, reduce heterogeneity and narrow confidence intervals. For every risk factor which had 3 or more CPRD studies in the “all dementia” category, we removed all but one CPRD paper per analysis. The CPRD paper spanning the longest time period was retained; where two studies had equal time spans, the study with the larger sample size was retained.

## Results

### Results of the searches

**Fig 1:**
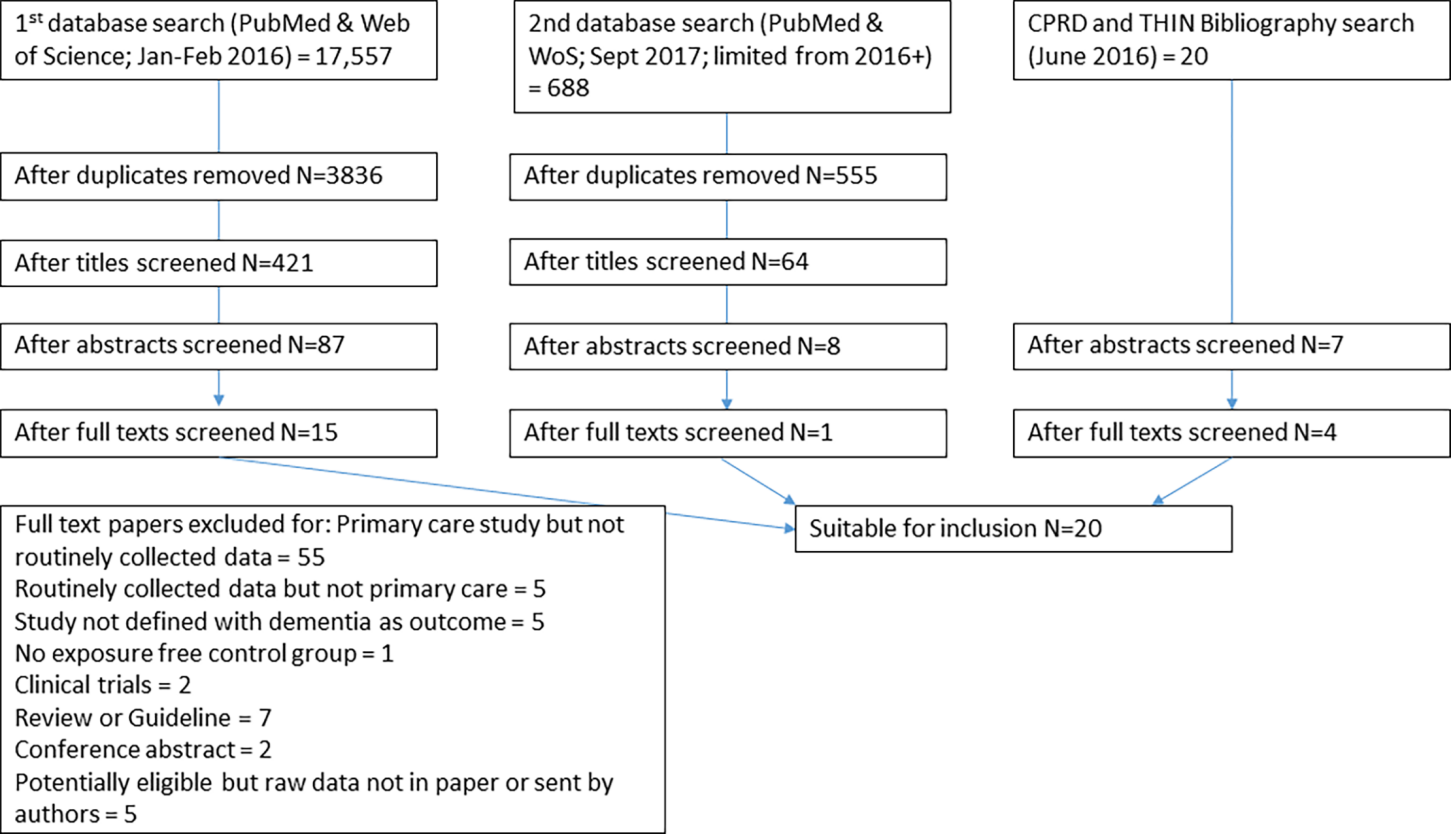
PRISMA flow diagram for study selection.

The initial database search identified 3,836 unique papers. Following title sifting, 421 abstracts from the database search, and 20 from searches of the CPRD bibliography and THIN publications list were examined, and of these, 94 papers were examined in full text. 19 papers met inclusion criteria and were included in the analysis. A second database search was performed which elicited 555 unique papers from 2016 onwards. Of these 64 abstracts were examined, and 8 papers in full text. One further paper was found to meet inclusion criteria. Thus 20 papers are included in the final analysis (Fig 1).

### Quality and Bias Assessment

Scoring is presented in S1 Table. There were 15 case-control studies all scoring 8 or 9 out of 10 stars on the Newcastle-Ottawa Scale. All 15 studies were categorized as low risk on all domains of the RoBANS, except for one study which had an unclear risk of bias in its handling of confounding variables. There were 5 cohort studies, of which one scored 7, three studies scored 8 and one study scored 9 on the Newcastle-Ottawa Scale. All 5 studies were categorized as low risk on all domains of the RoBANS, except for one study which had an unclear risk of bias in its handling of confounding variables. No studies were excluded on the basis of quality or bias.

We produced funnel plots for two comparisons: diabetes (12 studies) and hypertension (10 studies). Neither funnel plot showed asymmetry, suggesting the risk of publication bias was low (S1 Fig).

### Characteristics of studies

The characteristics of studies are shown in Table 1. Fourteen studies were based on UK data (12 from CPRD)[20, 25–37], three from the Netherlands,[18, 38, 39] two from Germany[40, 41] and one from Denmark.[42] Patients had a median follow up time of 5.25 years, the median age of participants was 80.6 years and the median proportion of females in the studies was 63%. Data for studies were recorded in primary care databases between 1990 and 2014. Study sizes ranged from 199 to 930,395 participants. More recent studies tended to have larger sample sizes and data extracts spanning longer periods.

**Table 1:** Study characteristics.

First Author name, date, country	Database	Extraction period	N-O score	RoBANS Summary	Type of dementia	Case definition	N Cases	N Controls	Matching Criteria	Conditions extracted	Medications extracted	Follow up time	Mean age (years)	% Female	Other details
Booker 2016, Germany [40]	Disease Analyzer database	2010–2014	8	Low Risk	NOS	ICD codes	11956	11956	Age, sex, physician and type of health insurance	Diabetes, Hypertension, obesity, hyperlipidemia, stroke, Parkinson’s disease, coronary heart disease, intracranial injury	Statins, proton-pump inhibitor, anti-hypertensive drugs	10 years	80.4	61%	
Buntix, 1996, Netherlands [39]	Registration Network of Family Practices	1993–1996	8	Low Risk	NOS	ICPC code	137	18966	Not matched -cohort	Old age Depression	-	1–10	Not given (>50y)	Not given	Cohort study, prevalence of dementia: 0.7%
Burton, 2013, UK [25]	Consultations in Primary Care Archive	2000–2008	8	Low Risk	NOS	Read code	400	1353	Age, sex, practice and consultation insame year	Anxiety, depression, cerebrovascular disease, diabetes, dyslipidemia, hypertension, hypotension, ischemic heart disease		3 years	Cases 81.40 (SD 6.5) Controls 80.87 (SD 6.19)	Cases62.0Controls 63.0	
Davies, 2011, UK [26]	CPRD	1997–2008	8	Low Risk	NOS	Read codes	9197	39166	Age (±5 years), practice, and sex	Coronary heart disease, diabetes, stroke	ARB, ACE-I, CC blocker, beta-blocker, thiazide diuretic	≤8 years	82.2 (6.9)	67.0	
Dregan, Chowienczyk, & Armstrong 2015, UK [36]	CPRD	1992–2014	8	Low Risk	AD, VaD and Lewy body	Read codes	31083 AD 23465 VaD, 1694 Lewy	350661	Age, sex, family practice and index date	Hypertension, BMI, smoking, alcohol, renal disease, CHD, stroke, diabetes, cancer, depression, COPD asthma, chronic inflammation	Antipsychotics, antidepressants, lipid lowering drugs, antihypertensive drugs, antidiabetes drugs	median 10.2 years	73	AD 67.0 VaD 59.0 Lewy 42.0 Controls 65.0	
Dregan, Chowienczyk & Gulliford, 2015, UK [37]	CPRD	2002–2013	8	Low Risk	NOS	Read codes	4183	458210	Age, sex and family practice	Inflammatory conditions e.g. Psoriasis, Crohn’s, Inflammatory arthritis		4 years average	82	67.0	Cohort study Dementia prevalence 0.9%
Dunn Holmes & Mullee, 2005, UK [33]	CPRD	1992–2002	8	Low Risk	All types combined	Read codes	9954	9374	Not matched, randomly sampled	Diabetes, smoking,	Lithium	Median 5.5 years	Cases 82, Controls 73	Cases 67.0 Controls 57.0	
Dunn, Mullee, Perry & Holmes, 2005, UK [27]	CPRD	1992–2002	9	Low Risk	NOS	Read codes	9954	9374	Not matched, randomly sampled	Diabetes, smoking, infective episodes	N prescriptions issued	4 years	Cases 82, Controls 73	Cases 67.0 Controls 57.0	Note both Dunn studies same sample
Imfeld, Bodmer, 2012, UK [29]	CPRD	1998–2008	9	Low Risk	AD	Read codes	7086	7086	Age, sex calendar time, GP and number of years recorded history in database	Smoking, BMI, Hypertension, dislipidemia, diabetes	Metformin sulfonylurea insulin thiazolidinedine	Median not given, over 10y in some cases	80.7 (SD 6.7)	69.0	
Imfeld, 2013, UK [34]	CPRD	1998–2008	9	Low Risk	AD or VD	Read codes	7086 AD case 4438 VD cases	11524 controls	Age, sex, calendar time, GP and number of years recorded history in database	Smoking, BMI, seizures		Median 1.5 years (IQR 0.5–3.0)	AD 80.7 [+-6.7] VaD 82.2 [+-6.6].	69.0 AD 59.0 VaD	
Imfeld, Pernus, 2013, UK [35]	CPRD	1998–2008	9	Low Risk	AD or VD	Read codes with algorithm	7086 AD case 4438 VD cases	7086 + 4438 controls	Age, sex, calendar time GP and number of years recorded history in database	Smoking, BMI, depression, diabetes, ischaemic heart disease, hypertension, hypercholesterolemia, atrial fibrillation, congestive heart failure, othostatic hypotension, COPD, inflammatory bowel disease osteporosis	ACE inhibitors, AT-II antagonists, beta-blockers, calcium channel blockers, diuretics, vasodilators, anti-arrhythmics, oral antidiabetics, insulin, statins, antiplatelets, anticoagulants, antiosteoporotics, intestinal anti-inflammatory agents, corticosteroids, NSAIDs, thyroid gland therapeutics, anticonvulsants, antidepressants, antipsychotics, benzodiazapines	Not given	Age distribution given	69.0 AD 59.0 VaD	Same population as above Imfeld 2012 and Imfeld 2013
Imfeld, 2015, UK [28]	CPRD	1998–2013	9	Low Risk	AD and VaD	Read codes for dementia or drug for AD, recording of specific dementia test, referral, brain imaging, or symptoms	16823 AD cases, 9636 VaD cases	16823 for AD 9636 for VaD	Age, sex, calendar time, general practice, and number of years of recorded history in database	BMI smoking, arterial hypertension, diabetes, dyslipidemia, atrial fibrillation, heart failure, depression	Antihypertensives, statins, platelet aggregation inhibitors anti-coagulents	Cases 8.0 years [3.0–12.5] controls 7.9 y [3.2–12.6]	78.8 AD 79.6 VaD	68.4 AD 60.9 VaD	Induction time—diagnosis shifted to earliest symptom, 2 years for AD and 3 years for VaD
Imfeld, 2016, UK [30]	CPRD	1998–2013	9	Low Risk	AD	Read codes for dementia or drug for AD, recording of specific dementia test, referral, brain imaging, or symptoms	19,463	19,463	Age, sex, calendar time, general practice, and number of years of recorded history in database	BMI, smoking, arterial hypertension, diabetes, dyslipidemia, atrial fibrillation, heart failure, asthma, COPD, inflammatory bowel disease, rheumatoid arthritis, psoriasis depression, influenza	corticosteroids, antibiotics	>3 years	Age distribution given	68.6	Same extraction specification as all Imfeld studies.
Jick, 2000, UK [32]	CPRD	1992–1998	8	Low Risk	NOS	Read codes	284	1080	Age, sex, calendar time, general practice, and number of years of recorded history in database	BMI, smoking, coronary artery disease, diabetes, transient cerebral ischemia, hypertension, coronary artery bypass surgery	Oestrogen, Statins	>4 years	Age distribution given	60.0	
Kessing, 2008, Denmark [42]	Danish population based register	1995–2005	7	Low Risk	NOS	ICD -10 codes	48483	1454977	Not matched -cohort		Lithium	Not available	52.7y At inclusion	63.5	Cohort study Dementia prevalence 3.2%
Koehler, 2015, Netherlands [38]	Dutch Registration Network of Family Practices	2000–2012	9	Low Risk	NOS	ICPC codes	1680	34111	Not matched -cohort	Depression		13 y observational period	Age distribution given	52.9	Cohort study dementia prevalence 4.6%
Ramakers, 2007, Netherlands [18]	Dutch Registration Network of Family Practices	1996–1999	8	Low Risk	NOS	Not clear, based on DSM-III R	74	125	Age, sex and practice	Education hypertension, CHD, stroke/TIA, cognitive symptoms, affective symptoms, behavioural symptoms, vascular symptoms, gait disturbances, changes in weight or appetite.		5 years	79	62.0	
Seshadri, 2001, UK [31]	CPRD	1990–1998	8	Low Risk	AD	Read codes, tests, evidence of progression	59	221	Age, Practice, index date, date of first prescription	BMI, smoking, hypercholesterolemia, hypertension, diabetes, ischemic heart disease,	Oestrogen replacement therapy	≥5 years	66.7	100	
Wagner, 2012, Germany [41]	Disease analyser database, Frankfurt	2003–2008	8	Low Risk	NOS	ICD-10 codes	1297	1297	Age, sex and index date	Parkinson’s, hypertension, myocardial infarction, cardiac insufficiency, schizophrenia, stroke, cancer, hepatic disorders, renal insufficiency, diabetes, lipid disorders	Cholesterol lowering agents NOS	3 years	80.6 (8.6)	62.0	
Walters, 2016, UK [20]	THIN	2000–2011	8	Low Risk	NOS	Read codes	13121	917274	Not matched -cohort	History of alcohol problem, anxiety, depression, CHD, stroke or TIA, atrial fibrillation, diabetes, Townsend score, smoking	Anti-hypertensive drugs, statins, hypnotics,	5 years	Age distribution given	Age 60–79 52.0 80+ 66.0	Cohort study dementia prevalence 1.4%

NOS: not otherwise specified. AD: Alzheimer’s Disease. VaD: Vascular Dementia. Unk: Unknown or not estimable. CPRD = clinical practice research datalink; THIN = The Health Improvement Network; ICPC = International Classification of Primary Care; ICD = International Classification of Diseases.

### Associations between risk factors and dementia

All associations between dementia and other conditions are shown in Table 2. Forest plots for comparisons with 4 or more studies can be seen in S2 Fig. Results are described by type of predictor, categorised as lifestyle, neuropsychiatric, cardiovascular, other conditions, and cognitive symptoms.

**Table 2:** Meta-analysis for lifestyle factors and conditions.

	All Dementia						Alzheimer’s Disease Only						Vascular Dementia Only					
Predictor	N studies	N cases	N controls	OR	95%CI	I^2^ Fixed/ Random	N studies	N cases	N controls	OR	95%CI	I^2^ Fixed/ Random	N studies	N cases	N controls	OR	95%CI	I^2^ Fixed/ Random
**Lifestyle and Demographics**																		
BMI > = 30	7	125987	421364	0.63*	0.42–0.94	100% R	5	74514	394254	0.54 *	0.33–0.90	100% R	3	37539	364735	**0.99**	0.70–1.39	98% R
Current Smoker	8	136157	1276116	**1.12**	0.89–1.41	99% R	5	74514	394254	**0.94**	0.83–1.07	90% R	3	37539	364735	1.4 ***	1.35–1.45	0% F
History of Alcohol Problem	1	13121	917274	0.14 ***	0.12–0.16	N/A	0						0					
Current Alcohol Usage	1	56424	350661	1.21***	1.19–1.24	N/A	1	31083	350661	1.21 ***	1.18–1.24	N/A	1	23465	350661	1.21 ***	1.18–1.25	N/A
Most deprived quintile Townsend deprivation index	2	67937	1123069	**1.04**	0.74–1.46	99% R	0	-	-	-	-	-	0	-	-	-	-	-
Education	1	74	125	**1.29**	0.63–2.65	N/A	0	-	-	-	-	-	0	-	-	-	-	-
**Neuropsychiatric**																		
Referral to psychiatrist, geriatrician or neurologist	1	50439	50439	38.83 ***	36.62–41.17	N/A	0	-	-	-	-	-	0	-	-	-	-	-
Depression	8	129029	1379811	1.64 ***	1.49–1.81	95% R	4	74455	394033	1.40***	1.29–1.53	91%R	2	33101	360297	1.64 ***	1.42–1.89	91% R
Anxiety	2	13521	981627	2.05 **	1.29–3.28	92% R	0	-	-	-	-	-	0	-	-	-	-	-
Epilepsy or Seizures	2	36067	81958	**3.79**	0.93–15.53	N/A	1	17178	35217	7.14 ***	4.69–10.87	N/A	1	7365	35217	9.46 ***	5.99–14.91	N/A
Parkinson's disease	2	13253	13253	1.94***	1.69–2.22	47%	0	-	-	-	-	-	0	-	-	-	-	-
Schizophrenia	1	1297	1297	**1.54**	0.86–2.76	N/A	0	-	-	-	-	-	0	-	-	-	-	-
**Cardiovascular risk factors**																		
Coronary or ischaemic heart disease	9	102857	1333360	**1.03**	0.88–1.21	98% R	3	38228	357968	0.76 ***	0.74–0.79	44% F	2	27903	355099	1.30 **	1.06–1.59	94% R
Myocardial infarction	1	1297	1297	**1.13**	0.78–1.64	N/A	0	-		-	-	-	0	-	-	-	-	-
Hypertension	10	127758	424139	**0.92**	0.82–1.03	97% R	5	74514	394290	0.78 **	0.66–0.93	98% R	3	37539	364735	1.16 ***	1.08–1.24	80% R
Hypotension	1	400	1353	**1.09**	0.53–2.25	N/A	0	-	-	-	-	-	0	-	-	-	-	-
Orthostatic hypotension	1	11524	11524	1.74***	1.48–2.04	N/A	1	7086	7086	1.59***	1.27–1.98	N/A	1	4438	4438	1.93***	1.52–2.45	N/A
Stroke, TIA or cerebrovascular disease	7	36329	972251	1.87 ***	1.41–2.49	98% R	1	31083	350661	0.55 ***	0.52–0.58	N/A	1	23465	350661	3.26***	3.14–3.37	N/A
Dyslipidemia	8	71412	73353	**1.02**	0.94–1.10	78% R	4	43431	43593	0.95**	0.92–0.99	2% F	2	14074	14074	1.17**	1.05–1.31	52% R
Atrial fibrillation	4	70567	974720	**1.18**	0.69–2.01	100% R	3	43372	43372	0.71 ***	0.68–0.75	0% F	2	14074	14074	1.65***	1.47–1.85	58% R
Heart failure and cardiac insufficiency	4	58743	58743	**0.88**	0.69–1.10	96% R	3	43372	43372	0.63***	0.60–0.67	0% F	2	14074	14074	1.28 ***	1.18–1.40	0% F
Coronary artery bypass	1	284	1080	**1.33**	0.61–2.86	N/A	0	-	-	-	-	-	0	-	-	-	-	-
Vascular symptoms^1^	1	74	125	**1.56**	0.70–3.50	N/A	0	-	-	-	-	-	0	-	-	-	-	-
**Chronic non-communicable**																		
Diabetes	12	159956	1389828	1.14 **	1.04–1.24	94% R	5	74514	394254	0.84 ***	0.78–0.90	76% R	2	33101	360297	1.51 ***	1.39–1.63	69% R
Renal disease or insufficiency	2	57539	351958	1.25***	1.22–1.28	0%F	1	31083	350661	1.06 ***	1.03–1.09	N/A	1	23465	350661	1.53***	1.49–1.58	N/A
Hepatic disorders	1	1297	1297	1.35 *	1.01–1.81	N/A	0	-	-	-	-	-	0	-	-	-	-	-
Cancer	2	57539	352858	**1.47**	0.60–3.61	98% R	1	31083	350661	0.89 ***	0.86–0.91	N/A	1	23465	650661	**1.00**	0.96–1.04	N/A
Osteoporosis	1	11524	11524	1.02	0.93–1.12	N/A	1	7086	7086	**0.99**	0.89–1.11	N/A	1	4438	4438	**1.06**	0.92–1.23	N/A
Thyroid Disorders	1	11524	11524	1.06	0.98–1.14	N/A	1	7086	7086	**0.97**	0.88–1.07	N/A	1	4438	4438	**1.22**	1.07–1.39	N/A
**Respiratory**																		
COPD w/wo Asthma	3	87229	381648	0.83**	0.74–0.94	89% R	3	57632	377210	0.76 ***	0.66–0.88	90% R	2	27903	355099	**0.99**	0.96–1.03	35% F
Asthma	1	19463	19463	0.85 ***	0.80–0.91	N/A	1	19463	19463	0.85 ***	0.80–0.91	N/A	0	-	-	-	-	-
**Injury**																		
Intracranial Injury	1	11956	11956	1.50**	1.15–1.94	N/A	0	-	-	-	-	-	0	-	-	-	-	-
**Inflammation and infection**																		
Infective episodes	1	9954	9374	1.30 ***	1.23–1.38	N/A	0	-	-	-	-	-	0	-	-	-	-	-
Influenza	1	19463	19463	**0.94**	0.94–1.01	N/A	1	19463	19463	**0.94**	0.94–1.01	N/A	0	-	-	-	-	-
Inflammatory conditions incl. bowel	4	91412	839858	**1.00**	0.97–1.03	0% F	3	57632	377210	0.96 *	0.91–1.00	0% F	2	27903	355099	**1.66**	0.68–4.04	98% R
Rheumatoid Arthritis	2	30987	30987	**0.92**	0.83–1.02	12% F	2	26549	26549	**0.89**	0.74–1.07	56% R	1	4438	4438	**0.97**	0.74–1.28	N/A
Psoriasis	1	19463	19463	**0.96**	0.96–1.06	N/A	1	19463	19463	**0.96**	0.96–1.06	N/A	0	-	-	-	-	-
**Dementia signs and symptoms**																		
Cognitive symptoms^2^	1	74	125	56.41***	16.41–193.95	N/A	0	-	-	-	-	-	0	-	-	-	-	-
Affective symptoms^3^	1	74	125	3.05 **	1.51–6.18	N/A	0	-	-	-	-	-	0	-	-	-	-	-
Behavioural symptoms^4^	1	74	125	7.76**	1.72–34.89	N/A	0	-	-	-	-	-	0	-	-	-	-	-
Gait disturbances^5^	1	74	125	6.11***	3.05–12.26	N/A	0	-	-	-	-	-	0	-	-	-	-	-
Change in weight or appetite	1	74	125	5.92***	2.21–15.80	N/A	0	-	-	-	-	-	0	-	-	-	-	-

1: Vascular symptoms defined as chest pains, loss of speech, temporary paralysis, continuous paralysis, loss of strength, decline in vision, and thick tongue

2: Cognitive symptoms defined as amnesia, forgetfulness, confusion, cognitive decline, orientation problems, language problems, problems with logical thinking, and loss of decorum

3: Affective symptoms defined as fatigue, irritability, anxiety, sleep-related problems, depressive mood, being upset, loss of initiative, loss of interest, crying, complaining, sadness, hyperventilation, mood changes, and suicidal ideation

4: Behavioural symptoms defined as restlessness, delusions/ hallucinations, aggression/agitation, changes in character, and suspicion

5: Gait disturbances defined as falls and problems with walking.

* P<0.05

** P<0.01

*** P<0.001

#### Lifestyle and demographics

Studies reported on current smoking, alcohol use, body mass index (BMI), deprivation index and level of education. High BMI (>30) had a negative association with all dementia (OR 0.63; 95%CI 0.42–0.94; 7 studies) and AD (OR 0.54; 95%CI 0.33–0.90; 5 studies) but no association with VaD (OR 0.99; 95% CI 0.70–1.39; 3 studies). Current smoking status showed no association with all dementia or AD but was significantly associated with VaD (OR 1.40; 95% CI 1.35–1.45; 3 studies). Current alcohol usage was positively associated with all types of dementia in one study (OR 1.21; 95%CI 1.19–1.24) but history of an alcohol problem was negatively associated with all cause dementia (OR 0.14; 90%CI 0.12–0.16; 1 study). Deprivation index and level of education were not associated with dementia.

#### Neuropsychiatric risk factors

Depression (OR 1.64; 95% CI 1.49–1.81; 8 studies), and anxiety (OR 2.05; 95% CI 1.29–3.28; 2 studies), showed strong positive associations with all dementia, as did a record of a referral to psychiatrist, geriatrician or neurologist (OR 38.83; 95% CI 36.62–41.17; 1 study). For depression, this association was maintained across AD and VaD. For epilepsy or seizures (OR 3.79; 95% CI 1.66–12.82; 2 studies) the positive effect found was non-significant for dementia NOS but significant for singles studies looking at AD (OR 7.14; 95% CI 4.69–10.87) and VaD (OR 9.46; 95% CI 5.99–14.91) dementia separately. A positive relationship was found between dementia and Parkinson’s disease (OR 1.94; 95%CI 1.69–2.22; 1 study) but not for schizophrenia (1 study).

#### Cardiovascular risk factors

Cardiovascular (CV) risk factors showed contrasting directions of association with AD and VaD. The only CV factors associated with all dementia were history of stroke (OR 1.87; 95% CI 1.41–2.49; 7 studies) and orthostatic hypotension (OR 1.74; 95% CI 1.48–2.04, 1 study). Most CV risk factors showed a negative relationship with AD, ranging from an OR of 0.55 (95%CI 0.52–0.58) for stroke (1 study) to 0.95 (95%CI 0.92–0.99) for dyslipidaemia (4 studies); the exception to this was orthostatic hypotension which showed a positive association (OR 1.59; 95%CI 1.27–1.98, 1 study). Most CV factors showed a positive association with VaD, ranging from OR 1.16 (95%CI 1.08–1.24) for hypertension (3 studies) to 3.26 (95%CI 3.14–3.37) for stroke (1 study).

#### Other conditions

Diabetes showed a positive association with all dementia (OR 1.14; 95%CI 1.04–1.22; 12 studies) and VaD (OR 1.51; 95%CI 1.39–1.63; 2 studies) and a negative association with AD (OR 0.84; 95%CI 0.78–0.90; 2 studies). Other associations with all dementia were found with renal disease (OR 1.25 95%CI 1.22–1.28; 2 studies), hepatic disorders (OR 1.35; 95% CI 1.01–1.81; 1 study), chronic obstructive pulmonary disorder (COPD) (OR 0.83; 95% CI0.74–0.94; 3 studies), and infective episodes (OR 1.30; 1.23–1.38; 1 study). Intracranial injury was also significantly associated with all dementia (OR 1.50; 95% CI 1.15–1.94; 1 study).

#### Dementia signs and symptoms

One small study (N = 199) examined composite variables of dementia signs and symptoms such as cognitive, affective and behavioural symptoms, gait disturbances and changes in weight or appetite. These all showed strong associations ranging from OR 3.05 (95%CI 1.51–6.18) for affective symptoms up to OR 56.41 (95%CI 16.41–193.95) for cognitive symptoms, in the year preceding a recorded dementia diagnosis.

### Associations between medication and dementia

Fewer studies reported on associations with medication, so many results in this section are from single studies. Results are reported in full in Table 3. Results are described by type of medication, categorised by neuropsychiatric, cardiovascular and diabetes, anti-inflammatory, and other types of medication.

**Table 3:** Meta-analysis for medications.

	All Dementia						Alzheimer’s Disease						Vascular Dementia					
Medication	N studies	N cases	N controls	OR	95%CI	I^2^ Fixed/ Random	N studies	N cases	N controls	OR	95%CI	I^2^ Fixed/ Random	N studies	N cases	N controls	OR	95%CI	I^2^ Fixed/ Random
N prescriptions issued	1	9954	9374	1.28***	1.20–1.37	N/A	0	-	-	-	-	-	0	-	-	-	-	-
**Neuro-psychiatric**																		
Antipsychotics	2	67766	362185	2.12 ***	1.82–2.46	91% R	2	38169	357747	1.89 ***	1.67–2.13	77% R	2	27903	355099	2.41 ***	1.97–2.95	89% R
Antidepressants	2	67766	362185	2.34 ***	2.06–2.67	93% R	2	38169	357747	2.07 ***	1.94–2.20	56% R	2	27903	355099	2.75 ***	2.11–3.57	95% R
Lithium	2	58437	1464351	1.42 *	1.01–1.99	65% R	0	-	-	-	-	-	0	-	-	-	-	-
Benzodiazapines	2	37983	37983	**1.17**	0.98–1.39	94% R	2	23909	23909	**1.04**	1.00–1.08	36% F	2	14074	14074	1.35 *	1.00–1.84	96% R
Hypnotics	1	13121	917274	2.18***	2.05–2.31	N/A	0	-	-	-	-	-	0	-	-	-	-	-
Anticonvulsants	1	11524	11524	1.64***	1.42–1.90	N/A	1	7086	7086	**1.06**	0.87–1.30	N/A	1	4438	4438	2.63***	2.11–3.28	N/A
**Cardiovascular**																		
Lipid lowering drugs incl Statins	6	108927	1308295	**1.02**	0.81–1.29	99% R	3	54992	374570	**0.89**	0.70–1.13	99% R	3	37539	364735	1.68 **	1.21–2.32	99% R
Antihypertensive drugs NOS	4	97119	1295691	**1.09**	0.80–1.55	100% R	2	47906	367484	**0.9**	0.52–1.57	100% R	2	33101	360297	**1.65**	0.91–2.99	100%R
Angiotensin receptor blockers	1	7427	461969	0.82 ***	0.75–0.88	N/A	0	-	-	-	-	-	0	-	-	-	-	-
ACE-I	2	20721	50690	0.87***	0.82–0.93	64% R	1	7086	7086	0.62***	0.57–0.68	N/A	1	4438	4438	1.25***	1.14–1.38	N/A
CC-blocker	2	20721	50690	0.85 ***	0.77–0.94	84% R	1	7086	7086	0.67***	0.61–0.73	N/A	1	4438	4438	**1.04**	0.94–1.14	N/A
beta-blocker	2	20721	50690	**0.90**	0.77–1.04	93% R	1	7086	7086	0.69***	0.64–0.75	N/A	1	4438	4438	**1.09**	0.98–1.20	N/A
AT-II antagonists	1	11524	11524	0.63***	0.56–0.70	N/A	1	7086	7086	0.69***	0.62–0.76	N/A	1	4438	4438	**0.93**	0.79–1.09	N/A
Vasodilators	1	11524	11524	**0.98**	0.91–1.06	N/A	1	7086	7086	0.69***	0.62–0.76	N/A	1	7086	7086	1.48***	1.32–1.65	N/A
Diuretics	2	20721	50690	0.81*	0.69–0.97	96% R	1	7086	7086	0.57 ***	0.53–0.61	N/A	1	4438	4438	1.11*	1.02–1.21	N/A
Anticoagulants	2	37983	37983	**0.99**	0.93–1.05	0% F	2	23909	23909	0.67***	0.62–0.73	0% F	2	14074	14074	1.54***	1.41–1.69	0% F
Antiplatelets	2	37983	37983	**1.21**	0.99–1.47	95% R	2	23909	23909	0.86 ***	0.82–0.90	28% F	2	14074	14074	1.93***	1.29–2.88	97% R
Anti-arrhythmics	1	11524	11524	**0.72**	0.62–0.85	N/A	1	7086	7086	0.60 ***	0.48–0.75	N/A	1	4438	4438	**0.91**	0.72–1.16	N/A
**Diabetes**																		
Anti-diabetes drugs NOS	2	67766	362185	**1.15**	0.99–1.35	87% R	2	38169	357747	0.89 ***	0.85–0.93	21% F	2	27903	355099	1.59***	1.30–1.95	85% R
Insulin	1	11524	11524	**0.98**	0.81–1.20	N/A	1	7086	7086	0.59 ***	0.44–0.79	N/A	1	4438	4438	1.64***	1.23–2.20	N/A
Metformin	1	7086	7086	0.80**	0.68–0.94	N/A	1	7086	7086	0.80**	0.68–0.94	N/A	0	-	-	-	-	-
Sulfonylurea	1	7086	7086	0.77***	0.66–0.90	N/A	1	7086	7086	0.77***	0.66–0.90	N/A	0	-	-	-	-	-
Thiazolidinedione	1	7086	7086	0.58**	0.38–0.89	N/A	1	7086	7086	0.58**	0.38–0.89	N/A	0	-	-	-	-	-
**Anti-inflammatories**																		
NSAIDS	3	125889	1279459	0.88***	0.85–0.91	55% R	1	7086	7086	0.87**	0.80–0.95	N/A	1	4438	4438	0.79***	0.71–0.89	N/A
Glucocorticoids	1	101244	350661	0.81***	0.79–0.82	N/A	0	-	-	-	-	-	0	-	-	-	-	-
Corticosteroids	2	30987	30987	0.73***	0.69–0.78	0% F	2	26549	26549	0.69***	0.61–0.79	69% R	1	4438	4438	**0.88**	0.76–1.02	N/A
Aspirin	1	13121	917274	2.15 ***	2.07–2.23	N/A	0	-	-	-	-	-	0	-	-	-	-	-
**Other**																		
Oestrogen replacement	2	343	1301	**1.06**	0.58–1.95	0% F	1	59	221	**1.08**	0.56–2.10	N/A	0	-	-	-	-	-
Antibiotics	1	19463	19463	0.89 ***	0.85–0.92	N/A	1	19463	19463	0.89 ***	0.85–0.92	N/A	0	-	-	-	-	-
Anti-osteoporotics	1	11524	11524	**1.00**	0.90–1.10	N/A	1	7086	7086	**0.97**	0.85–1.10	N/A	1	4438	4438	**1.04**	0.88–1.23	N/A
Intestinal anti-inflammatory agents	1	11524	11524	**1.02**	0.77–1.36	N/A	1	7086	7086	**0.8**	0.55–1.15	N/A	1	4438	4438	**1.52**	0.95–2.44	N/A
Thyroid gland therapeutics	1	11524	11524	**1.01**	0.92–1.10	N/A	1	7086	7086	**0.93**	0.84–1.04	N/A	1	4438	4438	**1.15**	1.00–1.13	N/A

* P<0.05

** P<0.01

*** P<0.001

#### Number of prescriptions issued

One study reported this risk factor finding a positive association between number of prescriptions and all dementia (OR 1.28; 95%CI 1.20–1.37).

#### Neurological and psychiatric medications

Antipsychotics (OR 2.12; 95%CI 1.82–2.46; 2 studies) and antidepressants (OR 2.34; 95%CI 2.06–2.67; 2 studies) showed the strongest associations with all dementia, with lithium, hypnotics, and anticonvulsants also showing positive associations.

#### Cardiovascular and diabetes medications

Positive associations were found between CV medications and VaD (e.g. anticoagulants OR 1.54 95%CI 1.30–1.95; 2 studies; and antiplatelets OR 1.93 95%CI 1.29–2.88; 2 studies), and negative associations with AD (e.g. anti-coagulants OR 0.67; 95%CI 0.62–0.73; 2 studies; anti-arrhythmics OR 0.60; 95%CI 0.48–0.75; 1 study). A similar pattern was found with diabetes medications (Table 3).

#### Anti-inflammatories

Non-steroidal anti-inflammatory drugs (NSAIDs) (with the exception of aspirin) and corticosteroids had a negative association with all dementia (NSAIDs OR 0.88; 95%CI 0.85–0.91; 3 studies; corticosteroids OR 0.73; 95% CI 0.69–0.73; 2 studies; aspirin OR 2.15; 95%CI 2.07–2.23; 1 study).

#### Other

Antibiotics had a negative association with all dementia (OR 0.89; 95%CI 0.85–0.92; 1 study).

### Sensitivity analysis

**Fig 2:**
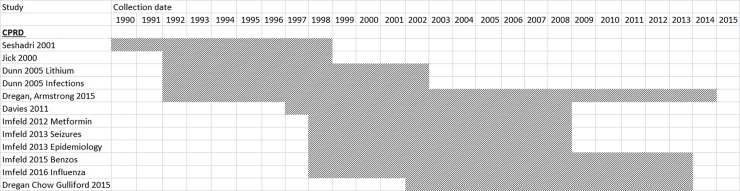
Data extraction periods for CPRD studies.

Twelve UK papers drew their samples from the same study population (CPRD). Fig 2 shows overlap between the data extraction periods for CPRD studies.

The meta-analysis was repeated with all but one CPRD study removed for every risk factor which had 3 or more CPRD studies in the all dementia category: BMI, smoking, depression, coronary heart disease, hypertension, dyslipidemia, atrial fibrillation, heart failure, diabetes, COPD, inflammatory conditions, and statins.

Results are presented in Table 4 and forest plots in S3 Fig. A reduction in the strength of association was found for high BMI (OR 1.09; 95% CI 0.93–1.28), all other risk factors retained their associations (or lack thereof).

**Table 4:** Sensitivity analysis results.

Predictor	N studies retained	CPRD study retained	N cases	N controls	OR	95%CI	I^2^ Fixed/ Random
BMI ≥ 30	2	Dregan 2015	68198	362617	**1.09**	0.93–1.28	88% R
Current Smoker	2	Dregan 2015	68414	1207995	**0.87**	0.45–1.67	100% R
Depression	5	Dregan 2015	71580	1322365	1.75 ***	1.48–2.07	95% R
Coronary heart disease	5	Dregan 2015	69896	1269634	**1.13**	0.89–1.45	99% R
Hypertension	5	Dregan 2015	58073	353657	**1.04**	0.83–1.17	88% R
Dyslipidemia	4	Imfeld 2015	40112	41065	**1.08**	0.99–1.17	71% R
Atrial Fibrillation	2	Imfeld 2015	39580	943733	**1.56**	0.65–3.72	100% R
Heart Failure	2	Imfeld 2015	27756	27756	**1.02**	0.74–1.41	93% R
Diabetes	5	Dregan 2015	71060	1270585	1.25***	1.16–1.35	83% R
COPD	1	Dregan 2015	56242	350661	0.86***	0.83–0.88	N/A
Inflammatory conditions including bowel	1	Dregan 2015	56242	350661	**1.00**	0.96–1.03	N/A
Statins	4	Dregan 2015	82616	1281188	**1.15**	0.87–1.51	99% R

* P<0.05

** P<0.01

*** P<0.001

## Discussion

This meta-analysis shows that a limited number of variables are associated with dementia diagnoses in primary care records, particularly neuropsychiatric and cardiovascular conditions, symptoms and medication. Cardiovascular risk factors were found to have strong negative associations with Alzheimer’s disease and strong positive associations with vascular dementia. Potential risk factors explored in this study will inform feature selection for development of a clinical risk tool for identifying or risk stratifying patients for dementia in primary care records.

Psychiatric or neurological risk factors were the category most positively associated across all dementia sub-types, with significant associations found for depression, anxiety, and seizures, as well as history of stroke. These have previously been reported to be associated with dementia.[43–45] There is some evidence that early dementia can manifest with anxiety [46, 47] and depression.[48, 49] Seizures are more commonly identified as sequelae of dementia [50]. Specifically “dementia-focussed” predictors such as cognitive and affective symptoms were examined in a single, small study (N = 199), and found to have very high association in the last year before diagnosis. In this study, only gait disturbances, such as falls, were predictive five years before diagnosis.[18]

Many cardiovascular risk factors appeared not to be associated with all-cause dementia, but when specific diagnoses were examined, they had contrasting associations between AD and VaD, which resulted in a non-association when these two outcomes were combined. Of note, several CV risk factors had a negative association with Alzheimer’s disease, as well as diabetes, cancer, COPD and asthma. This apparently “protective” effect is of interest. Previous studies have noted a positive relationship between CV risk factors and dementia,[51] even using them as positive predictors in a risk model,[52] although the association with AD appeared to be stronger when CV factors are measured at mid-life.[53] Other studies have noted an apparent protective effect of diseases such as cancer.[54] There are several possible explanations for this apparent protective effect. One possibility is that patients with these conditions are more likely to be diagnosed with VaD, rightly or wrongly, given that such disorders form part of the criteria for making a VaD diagnosis. This would reduce the pool of patients that can potentially be labelled with AD. One study showed this effect in diabetic patients.[55] A further possible explanation is that some physical conditions are under-managed or under-recorded by GPs when the patient has impaired cognitive functioning. Thus the reduction in apparent prevalence of these conditions in Alzheimer’s patients is due to recording or investigation biases rather than true differences. However, there may also be a biological protective effect, for example in cancer survivors, a genetic propensity against apoptosis might increase their risk of cancer while decreasing their risk of neurodegeneration.[54]

### Implications and future directions

This review and meta-analysis is designed to guide feature selection for creating a clinical risk tool for dementia in primary care. The types of risk factors consistently associated with dementia suggest that a patient record quality improvement model, or case detection algorithm, could feasibly be developed using primary care data, as the most predictive features include signs and symptoms of dementia that will have already been picked up by GPs. However, a risk stratification model, aiming to identify patients at increased risk of dementia when no clinical signs have yet been identified, may be more problematic, given the few clinical conditions consistently found to be associated with dementia.

Notably absent from the literature, despite a thorough examination of primary care database studies, were other elements in a primary care record which could be utilised in a predictive model for dementia. Patterns of attendance, missed appointments, missed prescriptions, and consultations by family members could all be used as indicators of behaviour change, and might increase the predictive validity of a model. The demonstration of their absence from the literature is a useful outcome of this review, and future research should undertake investigation as to whether these would be valuable predictors.

### Strengths and limitations

This is the first meta-analysis using routinely collected primary care data to establish a range of risk factors for dementia. We only included articles published in English, and only represented countries which have an established primary care database system. A single author extracted data from the included studies, which may have introduced error. We also only searched two medical literature databases, although we supplemented this with a targeted search of UK primary care database literature. This gives our work greater relevance to the UK but potentially limits the generalisability of our results beyond the UK primary care system.

We conducted a review of bias in individual studies, and we generated funnel plots to examine systematic heterogeneity for the best populated comparisons. We found little evidence of bias within studies, due to the data all being gathered in real time in routinely collected health records, and then extracted retrospectively, thus diminishing the risk of selection bias, detection bias, performance bias and attrition bias. However, there may have been a publication bias both in studies that were published (by only including significant associations in the results) and negative studies going unpublished. If this were the case, were we to recover these additional unpublished findings we would expect to find even fewer of the risk factors to show associations with the outcome.

Other characteristics of the data also urge cautious interpretation. First, heterogeneity was high for almost all analyses, which was unexpected due to the consistent nature of data generation between studies. This heterogeneity may have been caused by variation in the way participants, exposures and outcomes were defined in studies, such as the variation in average age of participant. The varying practices and coding systems in different countries may also have played a part as well as variation in coding practices of clinicians. For example, clinical entities were defined differently between studies, as some case definitions required a single Read code to define dementia and others a more complicated combination of codes. In many cases, code lists for the risk factors were not available. This may have limited the value of pooling results from studies. We attempted to mitigate the effects of heterogeneity by conducting random effects modelling for many analyses, but further thought may be needed to understand the source of this heterogeneity. Such heterogeneity may be a wider issue to be considered in all meta-analyses performed on routinely collected health record studies.

Another source of heterogeneity could have come from the timing of the measurement of exposures, as this varied between studies. In most studies, very little temporal information was given about when risk factors occurred or were measured, and the best description of our understanding is that they were present at some point in the record. More work is needed to unpick exactly when different risk factors have the greatest association with subsequent dementia, as longitudinal information and time-at-risk could usefully be incorporated into a clinical prediction model.

Additionally, the problem of resampling from the CPRD population meant that cases could not be assumed to be independent. However, our sensitivity analysis approach showed that for analyses with three or more CPRD studies, there were minimal differences between including one versus all CPRD studies, with the exception of BMI >30.

A further potential criticism of the overall endeavour of creating a risk prediction tool from primary care records is that GPs tend to diagnose dementia late in the course of the disease. Thus the data is not optimal for training an early risk identifier, as all studies assessing associations between risk factors and dementia will be identifying risk factors for late-diagnosed dementia on the whole. Additionally, those individuals who do get a diagnosis of dementia may differ systematically from the undiagnosed, and the low level of diagnosis in primary care records (possibly around 50% [11]) means that these differences could have substantial effects on our understanding of associations with various risk factors. As primary care records are human artefacts, created during the course of human interactions with only clinical purposes in mind, researchers must also be mindful of bias due to the underlying reasons or timings for recording, as well as choice of codes or free text, which may have multiple individual or systemic influences. Counter to this, the fact that the data is gathered prospectively in real time means that if the risk tool allows a long run up period, a wide collection of indicators will be found, including those linked to the early stages of the disease. Data collected in real time is also less biased by knowledge of the outcome. One future approach to mitigate the lack of coded diagnosis in the records would be to search for evidence of dementia or cognitive impairment in the textual, narrative, parts of the records, such as letters from specialists.

### Conclusions

This study provides a summary of the current literature on risk factors for dementia as ascertained from routinely collected primary care records. Few factors were consistently associated with dementia, and many gaps in the literature for examining potential risk factors were found. These findings are of potential value for feature selection for building a clinical risk prediction tool to aid the identification of cases of dementia in primary care.

## Supporting information

S1 Fig Funnel plots. (PDF)

S2 Fig Forest plots for main analysis. (PDF)

S3 Fig Forest plots for sensitivity analysis. (PDF)

S1 Table Quality and Bias Assessment scores. (PDF)

S2 Table PRISMA checklist. (DOC)

## References

[1] Prince M, Wimo A, Guerchet M, Ali G, Wu Y, Prina M, et al World Alzheimer Report 2015: The Global Impact of Dementia. 2015.

[2] Banerjee S. The Macroeconomics of Dementia—Will the World Economy Get Alzheimer's Disease? Archives of Medical Research. 2012;43(8):705–9. doi: 10.1016/j.arcmed.2012.10.006 23085453 10.1016/j.arcmed.2012.10.006

[3] Prince M, Bryce R, Ferr C. Alzheimer's Disease International World Alzheimer Report 2011: The benefits of early diagnosis and intervention. Institute of Psychiatry King's College London Alzheimer's Disease International 2011.

[4] Banerjee S, Wittenberg R. Clinical and cost effectiveness of services for early diagnosis and intervention in dementia. International journal of geriatric psychiatry. 2009;24(7):748–54. doi: 10.1002/gps.2191 19206079 10.1002/gps.2191

[5] Iliffe S, Manthorpe J, Eden A. Sooner or later? Issues in the early diagnosis of dementia in general practice: a qualitative study. Family Practice. 2003;20(4):376–81. 12876106 10.1093/fampra/cmg407

[6] Department of Health. Living well with dementia: A National Dementia Strategy 2009. Available from: https://www.gov.uk/government/uploads/system/uploads/attachment_data/file/168220/dh_094051.pdf.

[7] Department of Health. Prime Minister’s challenge on dementia: Delivering major improvements in dementia care and research by 2015 2012. Available from: https://www.gov.uk/government/uploads/system/uploads/attachment_data/file/215101/dh_133176.pdf.

[8] NHS England. Enhanced Service Specification: Dementia Identification Scheme 2014. Available from: http://www.england.nhs.uk/wp-content/uploads/2014/10/dementia-ident-schm-fin.pdf.

[9] Barrett E, Burns A. Dementia Revealed: What Primary Care Needs to Know: Hardwick CCG, NHS England, Department of Health, Royal College of General Practitioners; 2014 Available from: http://www.england.nhs.uk/wp-content/uploads/2014/09/dementia-revealed-toolkit.pdf.

[10] Pentzek M, Wollny A, Wiese B, Jessen F, Haller F, Maier W, et al Apart from nihilism and stigma: what influences general practitioners' accuracy in identifying incident dementia? The American journal of geriatric psychiatry: official journal of the American Association for Geriatric Psychiatry. 2009;17(11):965–75. Epub 2010/01/28. doi: 10.1097/JGP.0b013e3181b2075e . 20104054 10.1097/JGP.0b013e3181b2075e

[11] Connolly A, Gaehl E, Martin H, Morris J, Purandare N. Underdiagnosis of dementia in primary care: variations in the observed prevalence and comparisons to the expected prevalence. Aging & mental health. 2011;15(8):978–84. Epub 2011/07/23. doi: 10.1080/13607863.2011.596805 . 21777080 10.1080/13607863.2011.596805

[12] Stephan BCM, Tang E, Muniz-Terrera G. Composite risk scores for predicting dementia. Curr Opin Psychiatr. 2016;29(2):174–80. doi: 10.1097/yco.0000000000000235. WOS:000369605700012. 26779863 10.1097/YCO.0000000000000235

[13] Stephan BC, Kurth T, Matthews FE, Brayne C, Dufouil C. Dementia risk prediction in the population: are screening models accurate? Nat Rev Neurol. 2010;6 doi: 10.1038/nrneurol.2010.54 20498679 10.1038/nrneurol.2010.54

[14] Stephan BC, Brayne C. Risk factors and screening methods for detecting dementia: a narrative review. J Alzheimers Dis. 2014;42. 10.3233/JAD-141413 25261451

[15] Booth N. What are the Read Codes? Health Libr Rev. 1994;11(3):177–82. doi: 10.1046/j.1365-2532.1994.1130177.x 10139676 10.1046/j.1365-2532.1994.1130177.x

[16] CPRD. https://www.cprd.com/ [Accessed 6th June 2016].

[17] THIN. The Health Improvement Network https://www.ucl.ac.uk/pcph/research-groups-themes/thin-pub/publications [Accessed 6th June 2016].

[18] Ramakers IH, Visser PJ, Aalten P, Boesten JH, Metsemakers JF, Jolles J, et al Symptoms of preclinical dementia in general practice up to five years before dementia diagnosis. Dement Geriatr Cogn Disord. 2007;24(4):300–6. Epub 2007/08/25. doi: 10.1159/000107594 . 17717417 10.1159/000107594

[19] Jammeh E, Carroll C, Pearson S, Escudero J, Anastasiou A, Zajicek J, et al Using primary care data to identify undiagnosed dementia. J Neurol Neurosurg Psychiatry. 2015;86(11):e4-e. doi: 10.1136/jnnp-2015-312379.44

[20] Walters K, Hardoon S, Petersen I, Iliffe S, Omar RZ, Nazareth I, et al Predicting dementia risk in primary care: development and validation of the Dementia Risk Score using routinely collected data. BMC Medicine. 2016;14(1):1–12. doi: 10.1186/s12916-016-0549-y 26797096 10.1186/s12916-016-0549-y PMC4722622

[21] Moher D, Liberati A, Tetzlaff J, Altman DG. Preferred reporting items for systematic reviews and meta-analyses: the PRISMA statement. Ann Int Med. 2009;151(4):264–9. 19622511 10.7326/0003-4819-151-4-200908180-00135

[22] Park J, Lee Y, Seo H, Jang B, Son H, Kim S, et al., editors. Risk of bias assessment tool for non-randomized studies (RoBANS): development and validation of a new instrument. 19th Cochrane Colloquium; 2011.

[23] Stang A. Critical evaluation of the Newcastle-Ottawa scale for the assessment of the quality of nonrandomized studies in meta-analyses. Eur J Epidemiol. 2010;25(9):603–5. doi: 10.1007/s10654-010-9491-z 20652370 10.1007/s10654-010-9491-z

[24] Deeks J, Higgins J, Altman D, Green S. Cochrane handbook for systematic reviews of interventions version 5.1. 0 (updated March 2011). The Cochrane Collaboration. 2011.

[25] Burton C, Campbell P, Jordan K, Strauss V, Mallen C. The association of anxiety and depression with future dementia diagnosis: a case-control study in primary care. Family Practice. 2012;30(1):25–30. doi: 10.1093/fampra/cms044 22915794 10.1093/fampra/cms044 PMC3552314

[26] Davies NM, Kehoe PG, Ben-Shlomo Y, Martin RM. Associations of Anti-Hypertensive Treatments with Alzheimer's Disease, Vascular Dementia and Other Dementias. J Alz Dis. 2011;26:699–708. 10.3233/JAD-2011-110347 21709373

[27] Dunn N, Mullee M, Perry V, Holmes C. Association between dementia and infectious disease: evidence from a case-control study. Alzheimer Dis Assoc Disord. 2005;19(2):91–4. 15942327 10.1097/01.wad.0000165511.52746.1f

[28] Imfeld P, Bodmer M, Jick SS, Meier CR. Benzodiazepine Use and Risk of Developing Alzheimer's Disease or Vascular Dementia: A Case-Control Analysis. Drug safety. 2015 Epub 2015/07/01. doi: 10.1007/s40264-015-0319-3 . 26123874 10.1007/s40264-015-0319-3

[29] Imfeld P, Bodmer M, Jick SS, Meier CR. Metformin, Other Antidiabetic Drugs, and Risk of Alzheimer's Disease: A Population‐Based Case–Control Study. J Am Geriatr Soc. 2012;60(5):916–21. doi: 10.1111/j.1532-5415.2012.03916.x 22458300 10.1111/j.1532-5415.2012.03916.x

[30] Imfeld P, Toovey S, Jick SS, Meier CR. Influenza infections and risk of Alzheimer's disease. Brain, behavior, and immunity. 2016;57:187–92. Epub 2016/03/24. doi: 10.1016/j.bbi.2016.03.014 . 27006278 10.1016/j.bbi.2016.03.014

[31] Seshadri S, Zornberg GL, Derby LE, Myers MW, Jick H, Drachman DA. Postmenopausal estrogen replacement therapy and the risk of Alzheimer disease. Arch Neurol. 2001;58(3):435–40. 11255447 10.1001/archneur.58.3.435

[32] Jick H, Zornberg GL, Jick SS, Seshadri S, Drachman DA. Statins and the risk of dementia. Lancet. 2000;356:1627–31. 11089820 10.1016/s0140-6736(00)03155-x

[33] Dunn N, Holmes C, Mullee M. Does lithium therapy protect against the onset of dementia? Alzheimer Dis Assoc Disord. 2005;19(1):20–2. 15764867 10.1097/01.wad.0000155068.23937.9b

[34] Imfeld P, Bodmer M, Schuerch M, Jick SS, Meier CR. Seizures in patients with Alzheimer's disease or vascular dementia: A population-based nested case-control analysis. Epilepsia. 2013;54(4):700–7. doi: 10.1111/epi.12045 23215680 10.1111/epi.12045

[35] Imfeld P, Pernus YBB, Jick SS, Meier CR. Epidemiology, Co-Morbidities, and Medication Use of Patients with Alzheimer's Disease or Vascular Dementia in the UK. J Alzheimers Dis. 2013;35:565–73. doi: 10.3233/JAD-121819 23455986 10.3233/JAD-121819

[36] Dregan A, Chowienczyk P, Armstrong D. Patterns of anti‐inflammatory drug use and risk of dementia: a matched case–control study. Eur J Neurol. 2015;22(11):1421–8. doi: 10.1111/ene.12774 26177125 10.1111/ene.12774

[37] Dregan A, Chowienczyk P, Gulliford MC. Are inflammation and related therapy associated with all-cause dementia in a primary care population? J Alz Dis. 2015;46(4):1039–47. 10.3233/JAD-150171 26402631

[38] Köhler S, Buntinx F, Palmer K, Akker M. Depression, vascular factors, and risk of dementia in primary care: a retrospective cohort study. J Am Geriatr Soc. 2015;63(4):692–8. doi: 10.1111/jgs.13357 25900484 10.1111/jgs.13357

[39] Buntinx F, Kester A, Bergers J, Knottnerus JA. Is depression in elderly people followed by dementia? A retrospective cohort study based in general practice. Age and ageing. 1996;25(3):231–3. Epub 1996/05/01. . 8670559 10.1093/ageing/25.3.231

[40] Booker A, Jacob LE, Rapp M, Bohlken J, Kostev K. Risk factors for dementia diagnosis in German primary care practices. Int Psychogeriatr. 2016;28(7):1059–65. Epub 2016/01/09. doi: 10.1017/S1041610215002082 . 26744954 10.1017/S1041610215002082

[41] Wagner G, Icks A, Abholz HH, Schroder-Bernhardi D, Rathmann W, Kostev K. Antihypertensive treatment and risk of dementia: a retrospective database study. Int J Clin Pharmacol Ther. 2012;50(3):195–201. Epub 2012/03/01. . 22373832 10.5414/cp201284

[42] Kessing LV, Søndergård L, Forman JL, Andersen PK. Lithium treatment and risk of dementia. Archives of general psychiatry. 2008;65(11):1331–5. doi: 10.1001/archpsyc.65.11.1331 18981345 10.1001/archpsyc.65.11.1331

[43] Speck CE, Kukull WA, Brenner DE, Bowen JD, McCormick WC, Ten L, et al History of depression as a risk factor for Alzheimer's disease. Epidemiology. 1995;6(4):366–9. 7548342 10.1097/00001648-199507000-00006

[44] Ferretti L, McCurry SM, Logsdon R, Gibbons L, Teri L. Anxiety and Alzheimer's Disease. J Geriatr Psychiatry Neurol. 2001;14(1):52–8. doi: 10.1177/089198870101400111 11281317 10.1177/089198870101400111

[45] Sherzai D, Losey T, Vega S, Sherzai A. Seizures and dementia in the elderly: Nationwide Inpatient Sample 1999–2008. Epilepsy Behav. 2014;36:53–6. doi: 10.1016/j.yebeh.2014.04.015 24857809 10.1016/j.yebeh.2014.04.015

[46] Ballard C, Neill D, O’brien J, McKeith I, Ince P, Perry R. Anxiety, depression and psychosis in vascular dementia: prevalence and associations. Journal of affective disorders. 2000;59(2):97–106. 10837878 10.1016/s0165-0327(99)00057-9

[47] Seignourel PJ, Kunik ME, Snow L, Wilson N, Stanley M. Anxiety in dementia: a critical review. Clin Psychol Rev. 2008;28(7):1071–82. doi: 10.1016/j.cpr.2008.02.008 18555569 10.1016/j.cpr.2008.02.008 PMC2575801

[48] Cui X, Lyness JM, Tu X, King DA, Caine ED. Does depression precede or follow executive dysfunction? Outcomes in older primary care patients. The American journal of psychiatry. 2007;164(8):1221–8. Epub 2007/08/03. doi: 10.1176/appi.ajp.2007.06040690 . 17671285 10.1176/appi.ajp.2007.06040690

[49] Green RC, Cupples L, Kurz A, et al Depression as a risk factor for alzheimer disease: The mirage study. Arch Neurol. 2003;60(5):753–9. doi: 10.1001/archneur.60.5.753 12756140 10.1001/archneur.60.5.753

[50] Romanelli MF, Morris JC, Ashkin K, Coben LA. Advanced Alzheimer’s disease is a risk factor for late-onset seizures. Arch Neurol. 1990;47(8):847–50. 2375689 10.1001/archneur.1990.00530080029006

[51] Rosendorff C, Beeri MS, Silverman JM. Cardiovascular Risk Factors for Alzheimer's Disease. Am J Geriatr Cardiol. 2007;16(3):143–9. doi: 10.1111/j.1076-7460.2007.06696.x 17483665 10.1111/j.1076-7460.2007.06696.x

[52] Kivipelto M, Ngandu T, Laatikainen T, Winblad B, Soininen H, Tuomilehto J. Risk score for the prediction of dementia risk in 20 years among middle aged people: a longitudinal, population-based study. Lancet Neurol. 2006;5 doi: 10.1016/s1474-4422(06)70537-3 10.1016/S1474-4422(06)70537-3 16914401

[53] Kivipelto M, Ngandu T, Fratiglioni L, et al Obesity and vascular risk factors at midlife and the risk of dementia and alzheimer disease. Arch Neurol. 2005;62(10):1556–60. doi: 10.1001/archneur.62.10.1556 16216938 10.1001/archneur.62.10.1556

[54] Driver JA, Beiser A, Au R, Kreger BE, Splansky GL, Kurth T, et al Inverse association between cancer and Alzheimer’s disease: results from the Framingham Heart Study. Brit Med J. 2012;344 doi: 10.1136/bmj.e1442 22411920 10.1136/bmj.e1442 PMC3647385

[55] Beeri MS, Silverman JM, Davis KL, Marin D, Grossman HZ, Schmeidler J, et al Type 2 Diabetes Is Negatively Associated With Alzheimer's Disease Neuropathology. The Journals of Gerontology Series A: Biological Sciences and Medical Sciences. 2005;60(4):471–5. doi: 10.1093/gerona/60.4.471 10.1093/gerona/60.4.471 PMC3163091 15933386

