# Supplementary material for: Predicting dementia from primary care records: A systematic review and meta-analysis
Source: PLoS One. 2018 Mar 29;13(3):e0194735. doi: 10.1371/journal.pone.0194735 (PMC5875793; doi:10.1371/journal.pone.0194735)

S2 Figures: Funnel Plots

Figure 1: Funnel plot of comparison: Diabetes, outcome: Dementia NOS.

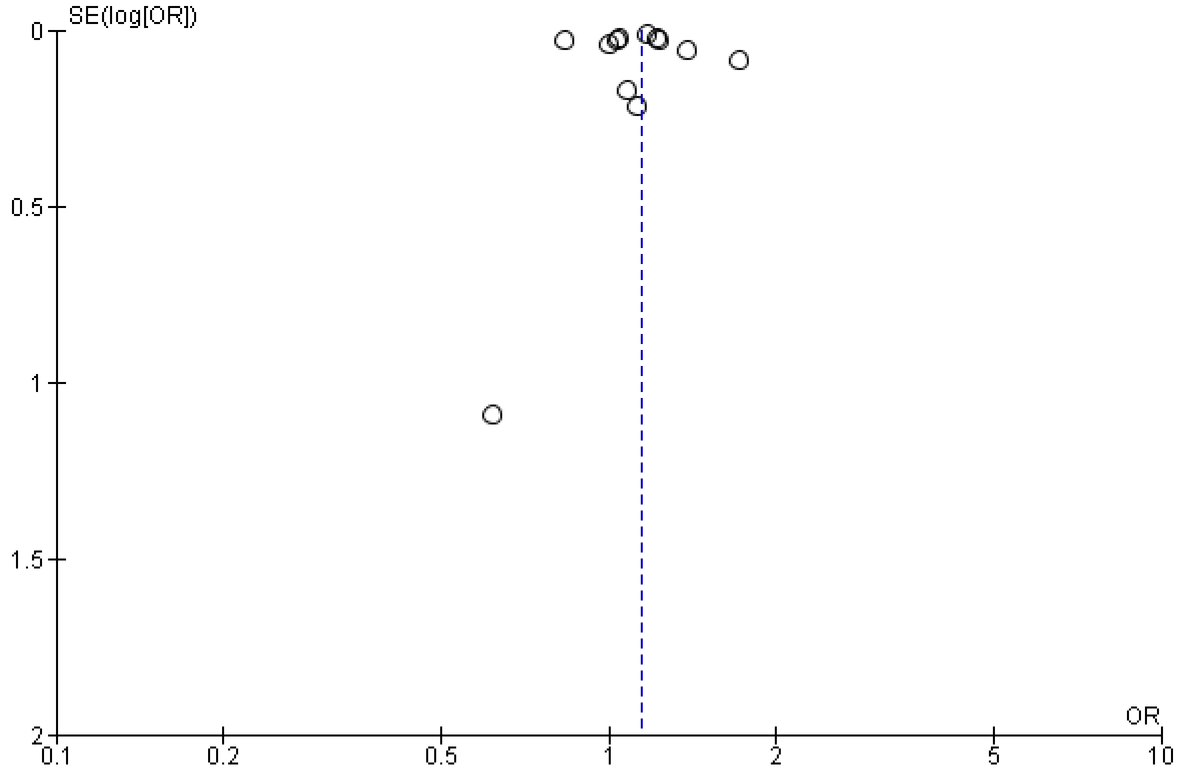

Figure 2: Funnel plot of comparison: Hypertension, outcome: Dementia NOS

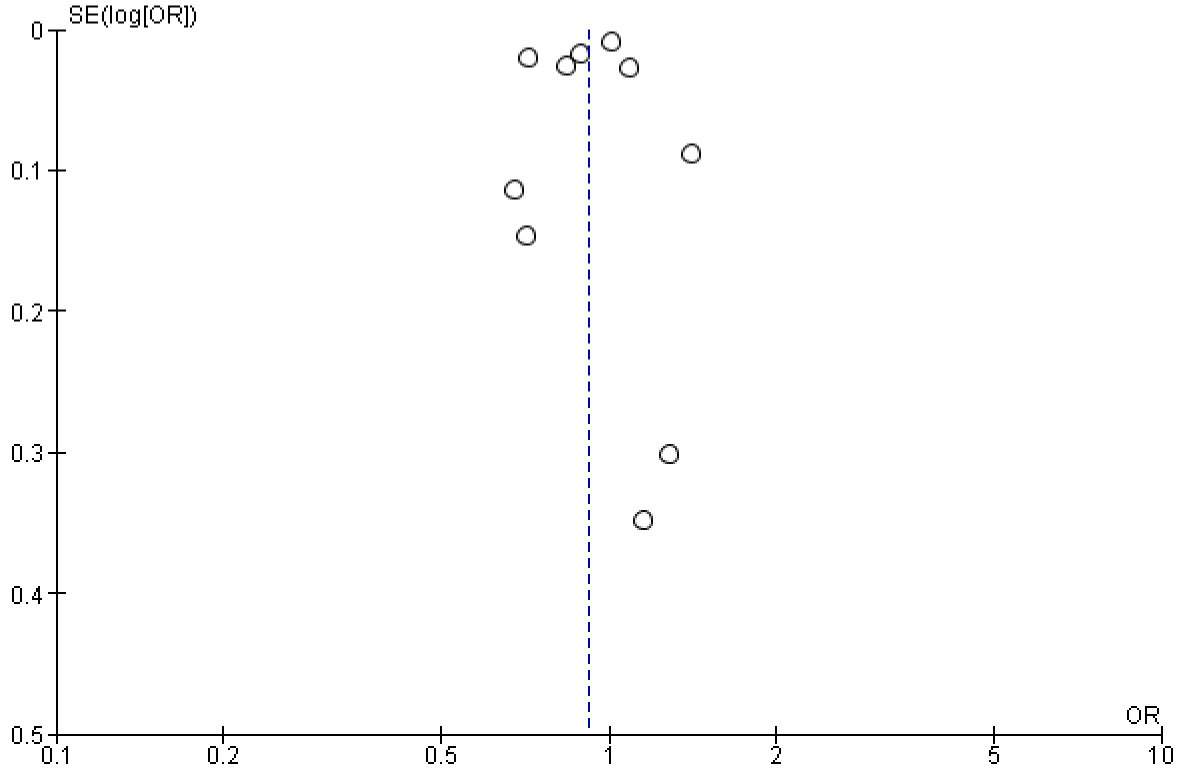

Supplement: S1 Fig — (PDF) [file pone.0194735.s001.pdf]
