# Supplementary material for: Predicting dementia from primary care records: A systematic review and meta-analysis
Source: PLoS One. 2018 Mar 29;13(3):e0194735. doi: 10.1371/journal.pone.0194735 (PMC5875793; doi:10.1371/journal.pone.0194735)

Figure 1: Forest plot of comparison: Obesity, outcome: Dementia NOS
Figure 2: Forest plot of comparison: Obesity, outcome: Alzheimer's
Figure 3: Forest Plot of comparison: Smoker, outcome: Dementia NOS
Figure 4: Forest plot of comparison: Smoker, outcome: Alzheimer's
Figure 5: Forest plot of comparison: Depression, outcome: Dementia NOS
Figure 6: Forest plot of comparison: Depression, outcome: Alzheimer's
Figure 7: Forest plot of comparison: Ischaemic Heart Disease, outcome: Dementia NOS
Figure 8: Forest Plot of comparison: Hypertension, outcome: Dementia NOS
Figure 9: Forest plot of comparison: Hypertension, outcome: Alzheimer's
Figure 10: Forest plot of comparison: Stroke, outcome: Dementia NOS
Figure 11: Forest plot of comparison: Dyslipidaemia, outcome: Dementia NOS
Figure 12: Forest plot of comparison: Dyslipidaemia, outcome: Alzheimer's NOS
Figure 13: Forest plot of comparison: Atrial Fibrillation, outcome: Dementia NOS
Figure 14: Forest plot of comparison: Heart Failure, outcome: Dementia NOS
Figure 15: Forest plot of comparison: Diabetes, outcome: Dementia NOS.
Figure 16: Forest plot of comparison: Diabetes, outcome: Alzheimer's
Figure 17: Forest plot of comparison: Inflammatory conditions, incl bowel, outcome: Dementia NOS
Figure 18: Forest plot of comparison: Lipid lowering drugs + statins, outcome: Dementia NOS
Figure 19: Forest plot of comparison: Anti-hypertensive drugs, outcome: Dementia NOS

Figure 1: Forest plot of comparison: Obesity, outcome: Dementia NOS

	Cases		Controls			Odds Ratio
**Study or Subgroup**	**Events**	**Total**	**Events**	**Total**	**Weight**	**M-H, Random, 95% CI**
Booker 2016	717	11956	717	11956	15.5%	1.00 [0.90, 1.11]
Dregan Chow 2015	6224	56242	33561	350661	15.7%	1.18 [1.14, 1.21]
Imfeld 2015 benzos	1003	11524	1520	11524	15.6%	0.63 [0.58, 0.68]
Imfeld 2016 influenza	5	59	55	221	8.2%	0.28 [0.11, 0.73]
Imfeld Pernus 2013	3169	26459	4132	26459	15.7%	0.74 [0.70, 0.77]
Jick 2000	32	284	214	1080	13.6%	0.51 [0.35, 0.76]
Seshadri 2001	1184	19463	3157	19463	15.6%	0.33 [0.31, 0.36]
**Total (95% CI)**		**125987**		**421364**	**100.0%**	**0.63 [0.42, 0.94]**
Total events	12334		43356			

Heterogeneity: Tau^2 = 0.26; Chi^2 = 1255.37, df = 6 ($P < 0.00001$); I^2 = 100%
Test for overall effect: Z = 2.27 (P = 0.02)

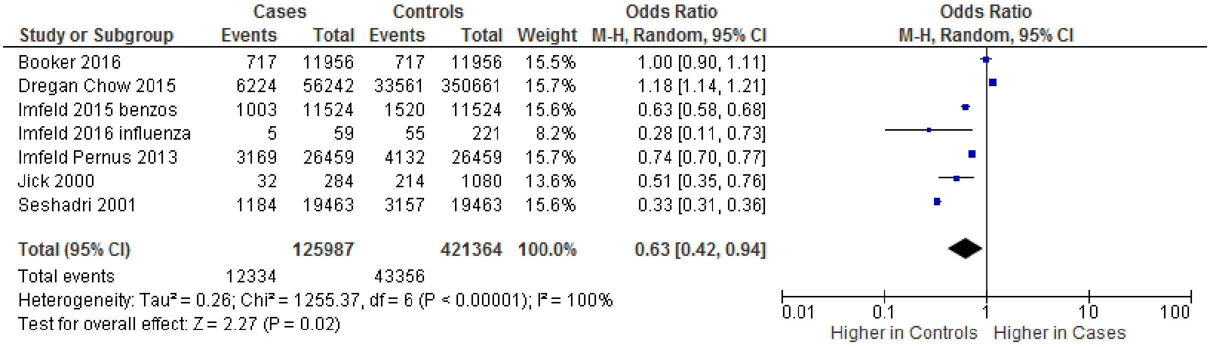

Figure 2: Forest plot of comparison: Obesity, outcome: Alzheimer's

	Cases		Control			Odds Ratio
**Study or Subgroup**	**Events**	**Total**	**Events**	**Total**	**Weight**	**M-H, Random, 95% CI**
Dregan Chow 2015	3114	31083	33561	350661	22.0%	1.05 [1.01, 1.09]
Imfeld 2015 benzos	1832	16823	2686	16823	22.0%	0.64 [0.60, 0.69]
Imfeld 2016 influenza	1184	19463	3157	19463	22.0%	0.33 [0.31, 0.36]
Imfeld Pernus 2013	564	7086	970	7086	21.8%	0.55 [0.49, 0.61]
Seshadri 2001	5	59	55	221	12.2%	0.28 [0.11, 0.73]
**Total (95% CI)**		**74514**		**394254**	**100.0%**	**0.54 [0.33, 0.90]**
Total events	6699		40429			

Heterogeneity: Tau^2 = 0.30; Chi^2 = 864.58, df = 4 ($P < 0.00001$); I^2 = 100%
Test for overall effect: Z = 2.38 (P = 0.02)

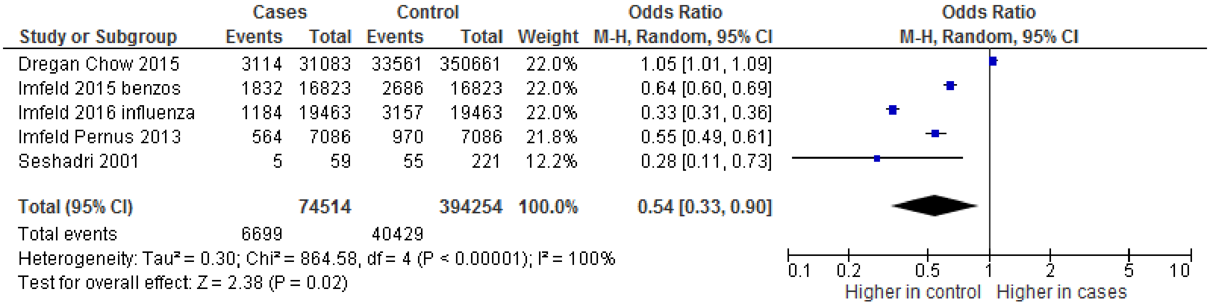

Figure 3: Forest Plot of comparison: Smoker, outcome: Dementia NOS

Study or Subgroup	Cases Events	Cases Total	Control Events	Control Total	Weight	Odds Ratio M-H, Random, 95% CI
Dregan Chow 2015	6314	56242	33181	350661	14.3%	1.21 [1.18, 1.25]
Dunn 2005 Lithium	41	284	85	1080	10.0%	1.98 [1.33, 2.94]
Imfeld 2015 benzos	2412	26459	2374	26459	14.2%	1.02 [0.96, 1.08]
Imfeld 2016 influenza	1518	19463	1652	19463	14.1%	0.91 [0.85, 0.98]
Imfeld Pernus 2013	1119	11524	1051	11524	14.0%	1.07 [0.98, 1.17]
Jick 2000	710	9954	395	9374	13.7%	1.75 [1.54, 1.98]
Seshadri 2001	10	59	32	221	5.4%	1.21 [0.55, 2.62]
Walters 2016	1468	12172	154468	857334	14.2%	0.62 [0.59, 0.66]
**Total (95% CI)**		**136157**		**1276116**	**100.0%**	**1.12 [0.89, 1.41]**
Total events	13592		193238			

Heterogeneity: Tau² = 0.09; Chi² = 531.42, df = 7 (P < 0.00001); I² = 99%
Test for overall effect: Z = 0.98 (P = 0.33)

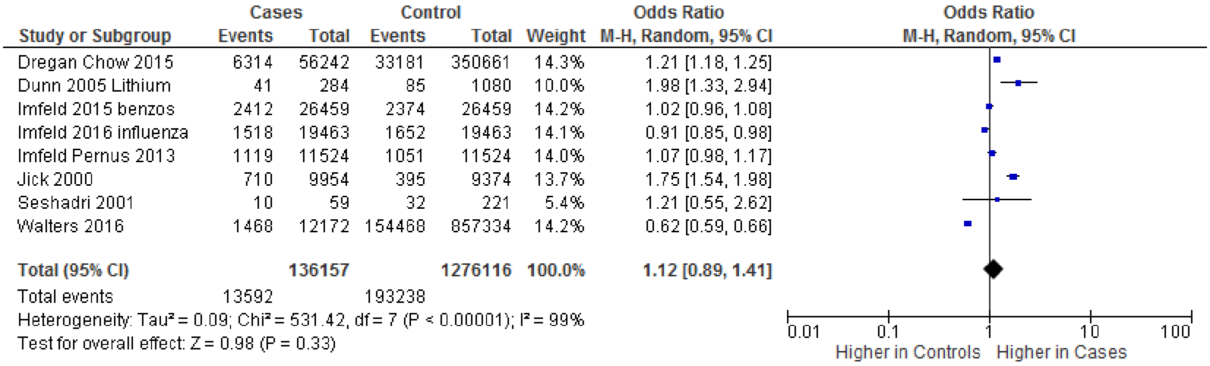

Figure 4: Forest plot of comparison: Smoker, outcome: Alzheimer's

Study or Subgroup	Cases Events	Cases Total	Control Events	Control Total	Weight	Odds Ratio M-H, Random, 95% CI
Dregan Chow 2015	3155	31083	33181	350661	26.3%	1.08 [1.04, 1.12]
Imfeld 2015 benzos	1349	16823	1546	16823	24.6%	0.86 [0.80, 0.93]
Imfeld 2016 influenza	1518	19463	1652	19463	24.8%	0.91 [0.85, 0.98]
Imfeld Pernus 2013	597	7086	669	7086	22.0%	0.88 [0.79, 0.99]
Seshadri 2001	10	59	32	221	2.4%	1.21 [0.55, 2.62]
**Total (95% CI)**		**74514**		**394254**	**100.0%**	**0.94 [0.83, 1.07]**
Total events	6629		37080			

Heterogeneity: Tau² = 0.02; Chi² = 41.21, df = 4 (P < 0.00001); I² = 90%
Test for overall effect: Z = 0.97 (P = 0.33)

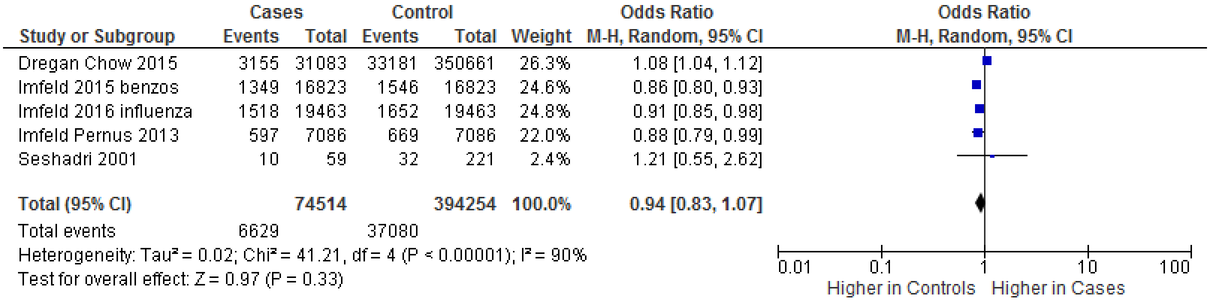

Figure 5: Forest plot of comparison: Depression, outcome: Dementia NOS

Study or Subgroup	Cases Events	Cases Total	Control Events	Control Total	Weight	Odds Ratio M-H, Random, 95% CI
Buntinx 1996	8	137	481	18966	1.7%	2.38 [1.16, 4.90]
Burton 2013	41	400	67	1353	4.4%	2.19 [1.46, 3.29]
Dregan Chow 2015	14492	56242	61804	350661	17.8%	1.62 [1.59, 1.66]
Imfeld 2015 benzos	4668	26459	3491	26459	17.3%	1.41 [1.34, 1.48]
Imfeld 2016 influenza	3995	19463	2796	19463	17.1%	1.54 [1.46, 1.62]
Imfeld Pernus 2013	2648	11524	1716	11524	16.6%	1.71 [1.59, 1.82]
Koehler 2015	58	1680	950	34111	7.7%	1.25 [0.95, 1.63]
Seshadri 2001	0	0	0	0		Not estimable
Walters 2016	2536	13121	98129	917274	17.4%	2.00 [1.91, 2.09]
**Total (95% CI)**		**129026**		**1379811**	**100.0%**	**1.64 [1.49, 1.81]**
Total events	28446		169434			

Heterogeneity: Tau² = 0.01; Chi² = 132.83, df = 7 (P < 0.00001); I² = 95%
Test for overall effect: Z = 9.84 (P < 0.00001)

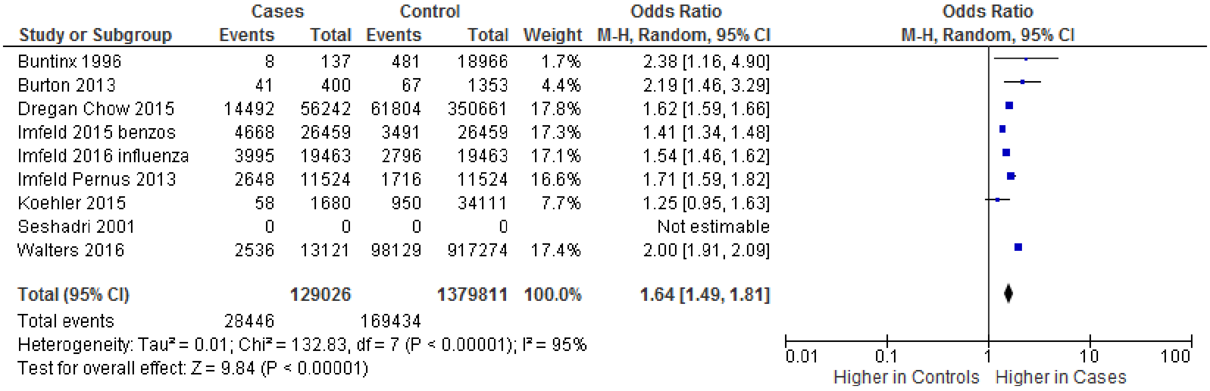

Figure 6: Forest plot of comparison: Depression, outcome: Alzheimer's

Study or Subgroup	Cases Events	Cases Total	Control Events	Control Total	Weight	Odds Ratio M-H, Random, 95% CI
Dregan Chow 2015	7567	31083	61804	350661	27.6%	1.50 [1.46, 1.55]
Imfeld 2015 benzos	2970	16823	2301	16823	25.0%	1.35 [1.28, 1.44]
Imfeld 2016 influenza	3995	19463	2796	19463	25.6%	1.54 [1.46, 1.62]
Imfeld Pernus 2013	1257	7086	1080	7086	21.8%	1.20 [1.10, 1.31]
**Total (95% CI)**		**74455**		**394033**	**100.0%**	**1.40 [1.29, 1.53]**
Total events	15789		67981			

Heterogeneity: Tau² = 0.01; Chi² = 33.26, df = 3 (P < 0.00001); I² = 91%
Test for overall effect: Z = 7.67 (P < 0.00001)

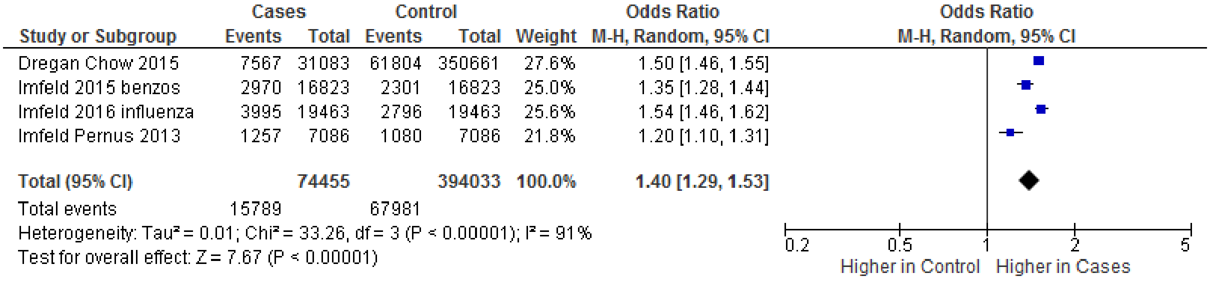

Figure 7: Forest plot of comparison: Ischaemic Heart Disease, outcome: Dementia NOS

Study or Subgroup	Cases Events	Cases Total	Control Events	Control Total	Weight	Odds Ratio M-H, Random, 95% CI
Booker 2016	3575	11956	3288	11956	14.8%	1.12 [1.06, 1.19]
Burton 2013	68	400	249	1353	9.9%	0.91 [0.68, 1.22]
Davies 2011	3106	9197	12938	39166	14.9%	1.03 [0.99, 1.08]
Dregan Chow 2015	12087	56242	73387	350661	15.0%	1.03 [1.01, 1.06]
Imfeld Pernus 2013	2484	11524	2729	11524	14.7%	0.89 [0.83, 0.94]
Jick 2000	61	284	320	1080	9.5%	0.65 [0.48, 0.89]
Ramakers 2007	16	74	30	125	3.9%	0.87 [0.44, 1.74]
Seshadri 2001	6	59	20	221	2.3%	1.14 [0.44, 2.97]
Walters 2016	2545	13121	119053	917274	14.9%	1.61 [1.54, 1.69]
**Total (95% CI)**		**102857**		**1333360**	**100.0%**	**1.03 [0.88, 1.21]**
Total events	23948		212014			

Heterogeneity: Tau² = 0.04; Chi² = 394.11, df = 8 (P < 0.00001); I² = 98%
Test for overall effect: Z = 0.34 (P = 0.73)

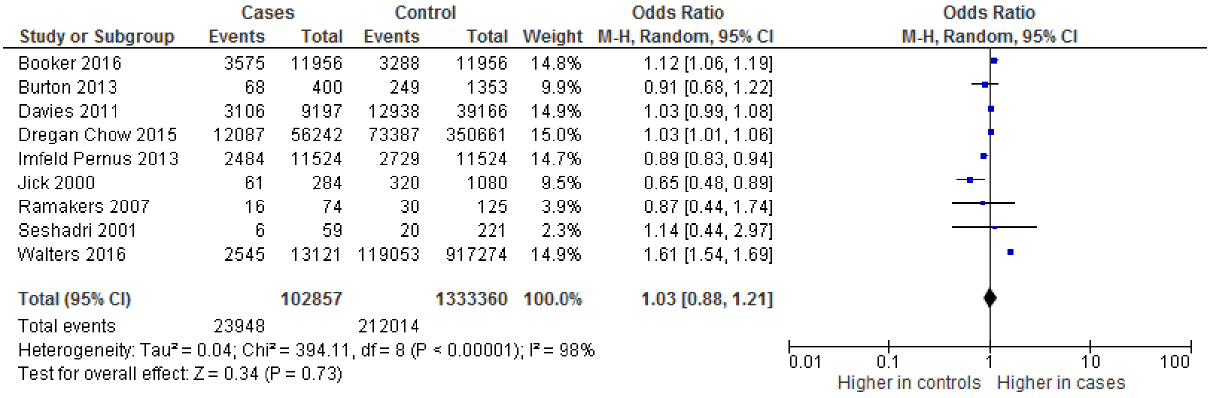

Figure 8: Forest Plot of comparison: Hypertension, outcome: Dementia NOS

Study or Subgroup	Cases Events	Cases Total	Control Events	Control Total	Weight	Odds Ratio M-H, Random, 95% CI
Booker 2016	7999	11956	7783	11956	13.4%	1.08 [1.03, 1.14]
Burton 2013	174	400	720	1353	9.0%	0.68 [0.54, 0.85]
Dregan Chow 2015	22794	56242	141606	350661	13.8%	1.01 [0.99, 1.02]
Imfeld 2015 benzos	12653	26459	13427	26459	13.7%	0.89 [0.86, 0.92]
Imfeld 2016 influenza	8761	19463	10358	19463	13.6%	0.72 [0.69, 0.75]
Imfeld Bodmer 2012	4926	11524	5424	11524	13.5%	0.84 [0.80, 0.88]
Jick 2000	80	284	385	1080	7.3%	0.71 [0.53, 0.94]
Ramakers 2007	31	74	45	125	2.9%	1.28 [0.71, 2.31]
Seshadri 2001	14	59	47	221	2.3%	1.15 [0.58, 2.28]
Wagner 2012	984	1297	895	1297	10.5%	1.41 [1.19, 1.68]
**Total (95% CI)**		**127758**		**424139**	**100.0%**	**0.92 [0.82, 1.03]**
Total events	58416		180690			

Heterogeneity: Tau² = 0.02; Chi² = 316.27, df = 9 (P < 0.00001); I² = 97%
Test for overall effect: Z = 1.50 (P = 0.13)

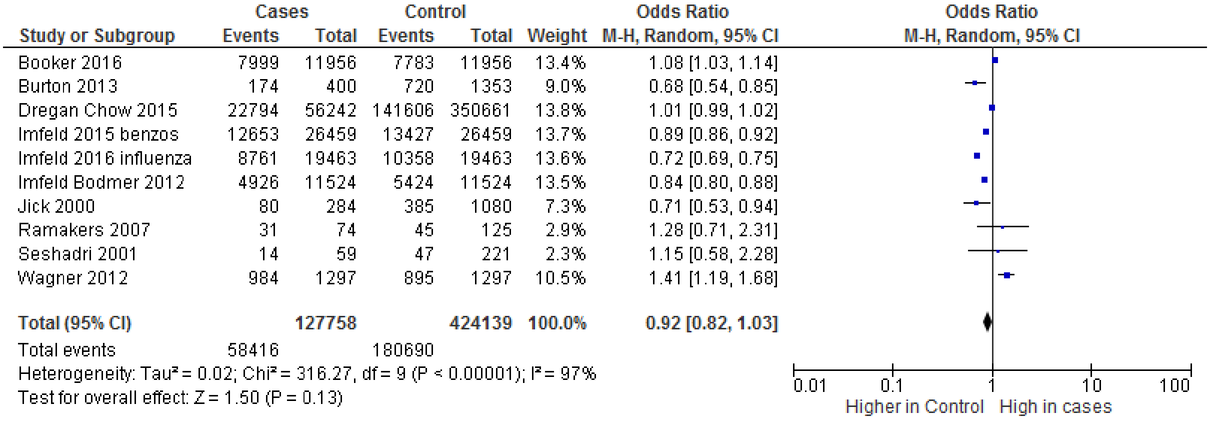

Figure 9: Forest plot of comparison: Hypertension, outcome: Alzheimer's

Study or Subgroup	Cases Events	Cases Total	Control Events	Control Total	Weight	Odds Ratio M-H, Random, 95% CI
Dregan Chow 2015	12218	31083	141606	350661	24.0%	0.96 [0.93, 0.98]
Imfeld 2015 benzos	7277	16823	8456	16823	23.8%	0.75 [0.72, 0.79]
Imfeld 2016 influenza	8761	19463	10358	19493	23.8%	0.72 [0.69, 0.75]
Imfeld Bodmer 2012	2627	7086	3345	7086	23.3%	0.66 [0.62, 0.70]
Seshadri 2001	14	59	47	227	5.1%	1.19 [0.60, 2.35]
**Total (95% CI)**		**74514**		**394290**	**100.0%**	**0.78 [0.66, 0.93]**
Total events	30897		163812			

Heterogeneity: Tau² = 0.03; Chi² = 244.62, df = 4 (P < 0.00001); I² = 98%
Test for overall effect: Z = 2.76 (P = 0.006)

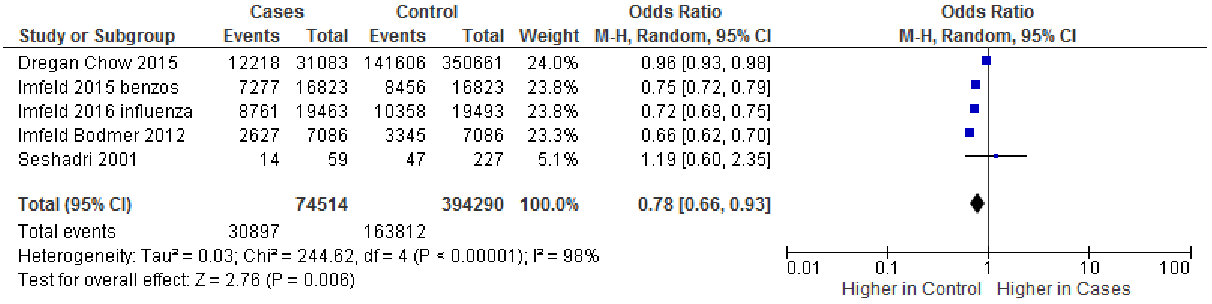

Figure 10: Forest plot of comparison: Stroke, outcome: Dementia NOS

Study or Subgroup	Cases Events	Cases Total	Control Events	Control Total	Weight	Odds Ratio M-H, Random, 95% CI
Booker 2016	2834	11956	1805	11956	17.0%	1.75 [1.64, 1.87]
Burton 2013	66	400	127	1353	14.1%	1.91 [1.38, 2.63]
Davies 2011	2415	9197	7091	39166	17.1%	1.61 [1.53, 1.70]
Jick 2000	34	284	97	1080	12.6%	1.38 [0.91, 2.09]
Ramakers 2007	13	74	14	125	7.1%	1.69 [0.75, 3.82]
Wagner 2012	186	1297	104	1297	15.1%	1.92 [1.49, 2.47]
Walters 2016	2145	13121	57052	917274	17.1%	2.95 [2.81, 3.09]
**Total (95% CI)**		**36329**		**972251**	**100.0%**	**1.87 [1.41, 2.49]**
Total events	7693		66290			

Heterogeneity: Tau² = 0.12; Chi² = 339.25, df = 6 (P < 0.00001); I² = 98%
Test for overall effect: Z = 4.35 (P < 0.0001)

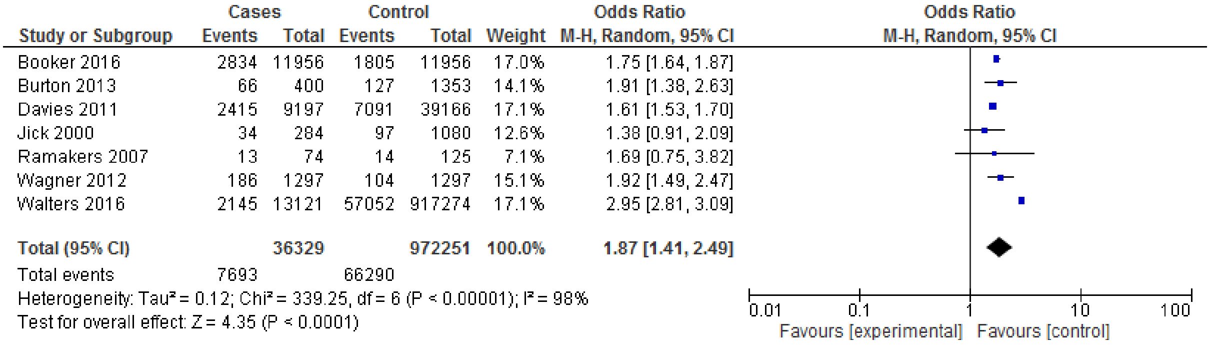

Figure 11: Forest plot of comparison: Dyslipidaemia, outcome: Dementia NOS

Study or Subgroup	Cases Events	Cases Total	Control Events	Control Total	Weight	Odds Ratio M-H, Random, 95% CI
Booker 2016	4304	11956	3993	11956	20.9%	1.12 [1.06, 1.18]
Burton 2013	43	400	200	1353	4.0%	0.69 [0.49, 0.99]
Imfeld 2015 benzos	4138	26459	3965	26459	21.4%	1.05 [1.00, 1.10]
Imfeld 2016 influenza	2998	19463	3096	19463	20.8%	0.96 [0.91, 1.02]
Imfeld Pernus 2013	1096	11524	1145	11524	17.9%	0.95 [0.87, 1.04]
Jick 2000	29	284	142	1080	2.9%	0.75 [0.49, 1.15]
Seshadri 2001	3	29	7	221	0.3%	3.53 [0.86, 14.48]
Wagner 2012	525	1297	475	1297	11.8%	1.18 [1.00, 1.38]
**Total (95% CI)**		**71412**		**73353**	**100.0%**	**1.02 [0.94, 1.10]**
Total events	13136		13023			

Heterogeneity: Tau² = 0.01; Chi² = 32.05, df = 7 (P < 0.0001); I² = 78%
Test for overall effect: Z = 0.46 (P = 0.65)

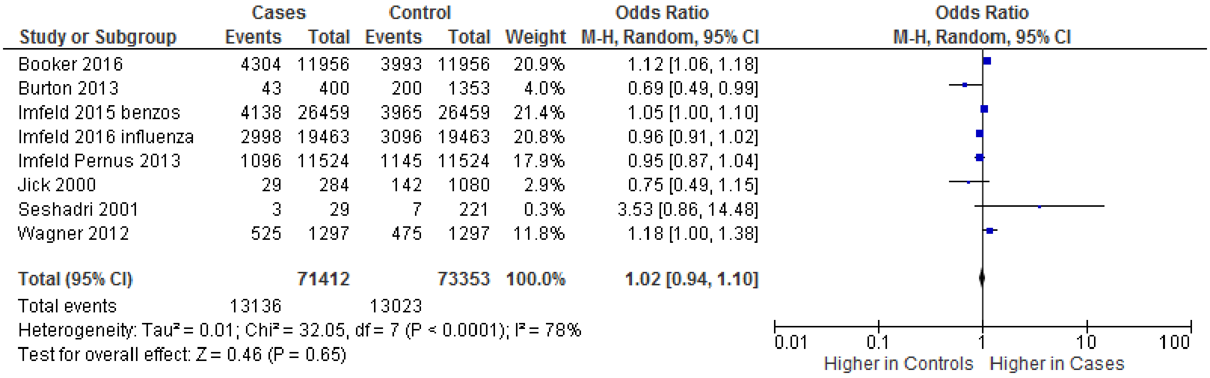

Figure 12: Forest plot of comparison: Dyslipidaemia, outcome: Alzheimer's NOS

Study or Subgroup	Cases Events	Cases Total	Control Events	Control Total	Weight	Odds Ratio M-H, Fixed, 95% CI
Imfeld 2015 benzos	2487	16823	2575	16823	40.1%	0.96 [0.90, 1.02]
Imfeld 2016 influenza	2998	19463	3096	19463	47.8%	0.96 [0.91, 1.02]
Imfeld Bodmer 2012	643	7086	726	7086	12.1%	0.87 [0.78, 0.98]
Imfeld Pernus 2013	0	0	0	0		Not estimable
Seshadri 2001	3	59	7	221	0.1%	1.64 [0.41, 6.54]
**Total (95% CI)**		**43431**		**43593**	**100.0%**	**0.95 [0.92, 0.99]**
Total events	6131		6404			

Heterogeneity: Chi² = 3.05, df = 3 (P = 0.38); I² = 2%
Test for overall effect: Z = 2.58 (P = 0.010)

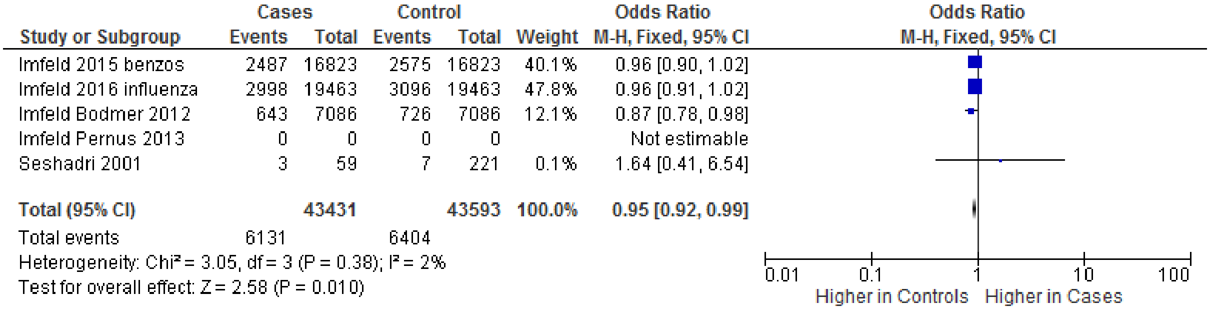

Figure 13: Forest plot of comparison: Atrial Fibrillation, outcome: Dementia NOS

Study or Subgroup	Cases Events	Cases Total	Control Events	Control Total	Weight	Odds Ratio M-H, Random, 95% CI
Imfeld 2015 benzos	2413	26459	2397	26459	25.0%	1.01 [0.95, 1.07]
Imfeld 2016 influenza	1615	19463	2157	19463	25.0%	0.73 [0.68, 0.78]
Imfeld Pernus 2013	1331	11524	1244	11524	25.0%	1.08 [0.99, 1.17]
Walters 2016	1241	13121	38040	917274	25.0%	2.41 [2.28, 2.56]
**Total (95% CI)**		**70567**		**974720**	**100.0%**	**1.18 [0.69, 2.01]**
Total events	6600		43838			

Heterogeneity: Tau² = 0.30; Chi² = 797.92, df = 3 (P < 0.00001); I² = 100%
Test for overall effect: Z = 0.59 (P = 0.56)

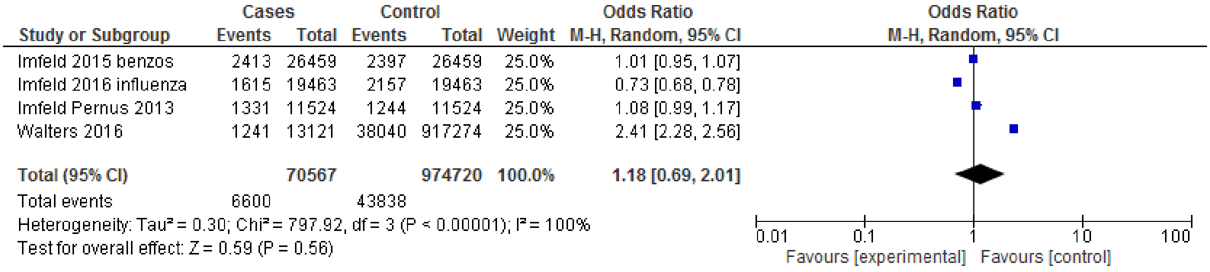

Figure 14: Forest plot of comparison: Heart Failure, outcome: Dementia NOS

Study or Subgroup	cases Events	cases Total	Control Events	Control Total	Weight	Odds Ratio M-H, Random, 95% CI
Imfeld 2015 benzos	1412	26459	1602	26459	25.7%	0.87 [0.81, 0.94]
Imfeld 2016 influenza	947	19463	1478	19463	25.5%	0.62 [0.57, 0.68]
Imfeld Pernus 2013	1048	11524	1142	11524	25.4%	0.91 [0.83, 0.99]
Wagner 2012	525	1297	465	1297	23.4%	1.22 [1.04, 1.43]
**Total (95% CI)**		**58743**		**58743**	**100.0%**	**0.88 [0.69, 1.10]**
Total events	3932		4687			

Heterogeneity: Tau² = 0.05; Chi² = 73.29, df = 3 (P < 0.00001); I² = 96%
Test for overall effect: Z = 1.13 (P = 0.26)

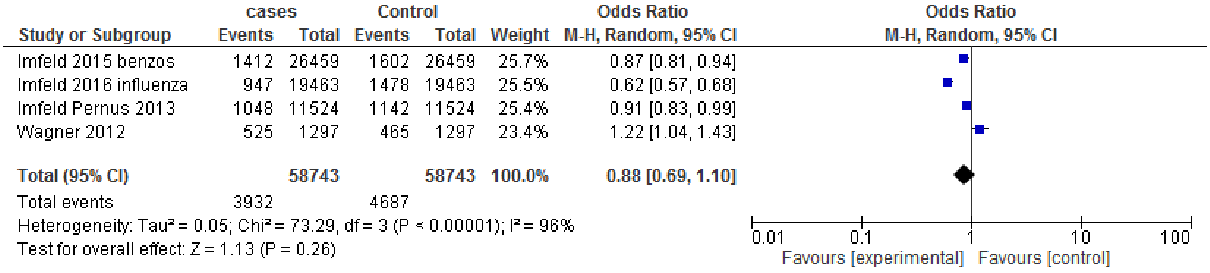

Figure 15: Forest plot of comparison: Diabetes, outcome: Dementia NOS.

Study or Subgroup	Cases Events	Cases Total	Control Events	Control Total	Weight	Odds Ratio M-H, Random, 95% CI
Booker 2016	4053	11956	3539	11956	10.8%	1.22 [1.15, 1.29]
Burton 2013	51	400	162	1353	4.2%	1.07 [0.77, 1.50]
Davies 2011	1363	9197	5629	39166	10.6%	1.04 [0.97, 1.11]
Dregan Chow 2015	8054	56242	43925	350661	11.2%	1.17 [1.14, 1.20]
Dunn 2005 Infections	772	9954	536	9374	9.5%	1.39 [1.24, 1.55]
Imfeld 2015 benzos	3084	26459	2979	26459	10.8%	1.04 [0.99, 1.10]
Imfeld 2016 influenza	2055	19463	2422	19463	10.7%	0.83 [0.78, 0.88]
Imfeld Bodmer 2012	1225	11524	1221	11524	10.2%	1.00 [0.92, 1.09]
Jick 2000	30	284	103	1080	3.0%	1.12 [0.73, 1.72]
Seshadri 2001	1	59	6	221	0.2%	0.62 [0.07, 5.23]
Wagner 2012	470	1297	322	1297	7.9%	1.72 [1.45, 2.04]
Walters 2016	1415	13121	81724	917274	10.8%	1.24 [1.17, 1.31]
**Total (95% CI)**		**159956**		**1389828**	**100.0%**	**1.14 [1.04, 1.24]**
Total events	22573		142568			

Heterogeneity: Tau² = 0.02; Chi² = 177.12, df = 11 (P < 0.00001); I² = 94%
Test for overall effect: Z = 2.91 (P = 0.004)

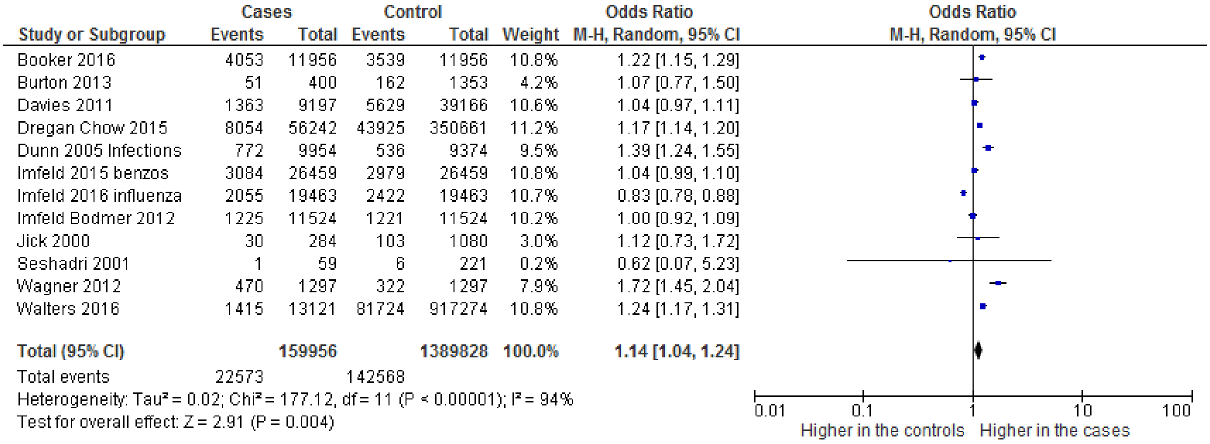

Figure 16: Forest plot of comparison: Diabetes, outcome: Alzheimer's

Study or Subgroup	Cases Events	Cases Total	Control Events	Control Total	Weight	Odds Ratio M-H, Random, 95% CI
Dregan Chow 2015	3573	31083	43925	350661	30.3%	0.91 [0.87, 0.94]
Imfeld 2015 benzos	1602	16823	1894	16823	25.1%	0.83 [0.77, 0.89]
Imfeld 2016 influenza	2055	19463	2422	19463	26.4%	0.83 [0.78, 0.88]
Imfeld Bodmer 2012	570	7086	747	7086	18.1%	0.74 [0.66, 0.83]
Seshadri 2001	1	59	6	221	0.1%	0.62 [0.07, 5.23]
**Total (95% CI)**		**74514**		**394254**	**100.0%**	**0.84 [0.78, 0.90]**
Total events	7801		48994			

Heterogeneity: Tau² = 0.00; Chi² = 16.42, df = 4 (P = 0.002); I² = 76%
Test for overall effect: Z = 4.83 (P < 0.00001)

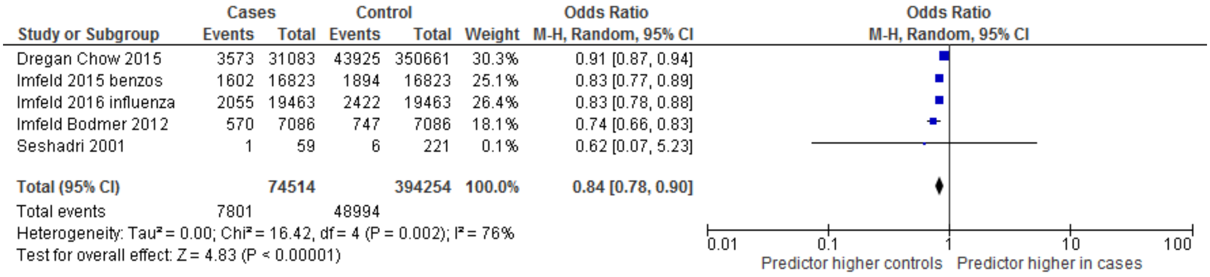

Figure 17: Forest plot of comparison: Inflammatory conditions, incl bowel, outcome: Dementia NOS

Study or Subgroup	Cases Events	Cases Total	Control Events	Control Total	Weight	Odds Ratio M-H, Fixed, 95% CI
Dregan Chow 2015	4096	56242	25581	350661	75.0%	1.00 [0.96, 1.03]
Dregan, Armstrong 2015	1378	4183	152172	458210	21.1%	0.99 [0.93, 1.05]
Imfeld 2016 influenza	215	19463	219	19463	2.5%	0.98 [0.81, 1.19]
Imfeld Pernus 2013	124	11524	122	11524	1.4%	1.02 [0.79, 1.31]
**Total (95% CI)**		**91412**		**839858**	**100.0%**	**1.00 [0.97, 1.03]**
Total events	5813		178094			

Heterogeneity: Chi² = 0.12, df = 3 (P = 0.99); I² = 0%
Test for overall effect: Z = 0.27 (P = 0.79)

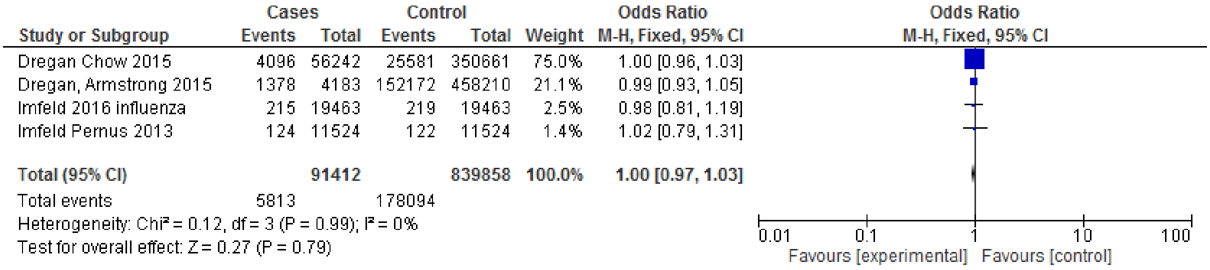

Figure 18: Forest plot of comparison: Lipid lowering drugs + statins, outcome: Dementia NOS

Study or Subgroup	Experimental Events	Experimental Total	Control Events	Control Total	Weight	Odds Ratio M-H, Random, 95% CI
Booker 2016	4340	11956	4065	11956	15.8%	1.11 [1.05, 1.17]
Dregan Chow 2015	17332	56242	79682	350661	16.0%	1.51 [1.49, 1.54]
Imfeld 2015	7451	26459	7255	26459	15.9%	1.04 [1.00, 1.08]
Imfeld Pernus 2013	2441	11524	2451	11524	15.8%	0.99 [0.93, 1.06]
Jick 2000	12	284	100	1080	6.4%	0.43 [0.23, 0.80]
Wagner 2012	343	1297	324	1297	14.2%	1.08 [0.91, 1.29]
Walters 2016	2272	13121	165549	917274	15.9%	0.95 [0.91, 1.00]
**Total (95% CI)**		**120883**		**1320251**	**100.0%**	**1.04 [0.85, 1.27]**
Total events	34191		259426			

Heterogeneity: Tau² = 0.06; Chi² = 671.42, df = 6 (P < 0.00001); I² = 99%
Test for overall effect: Z = 0.36 (P = 0.72)

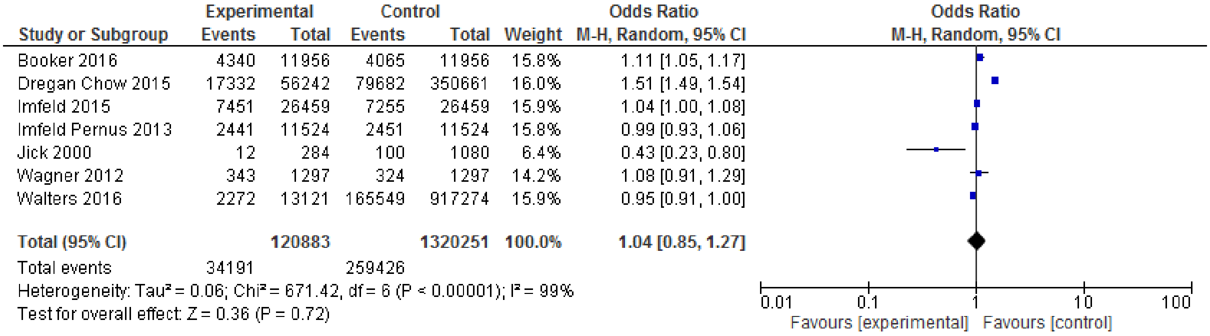

Figure 19: Forest plot of comparison: Anti-hypertensive drugs, outcome: Dementia NOS

	Experimental		Control			Odds Ratio
**Study or Subgroup**	**Events**	**Total**	**Events**	**Total**	**Weight**	**M-H, Random, 95% CI**
Booker 2016	9971	11956	9660	11956	20.2%	1.19 [1.12, 1.28]
Dregan Chow 2015	32926	56242	167660	350661	20.4%	1.54 [1.51, 1.57]
Imfeld 2015	15537	26459	16676	26459	20.3%	0.83 [0.81, 0.86]
Wagner 2012	336	1297	375	1297	18.8%	0.86 [0.72, 1.02]
Walters 2016	5434	13121	327546	917274	20.3%	1.27 [1.23, 1.32]
**Total (95% CI)**		**109075**		**1307647**	**100.0%**	**1.11 [0.85, 1.46]**
Total events	64204		521917			

Heterogeneity: Tau² = 0.09; Chi² = 980.49, df = 4 (P < 0.00001); I² = 100%
Test for overall effect: Z = 0.79 (P = 0.43)

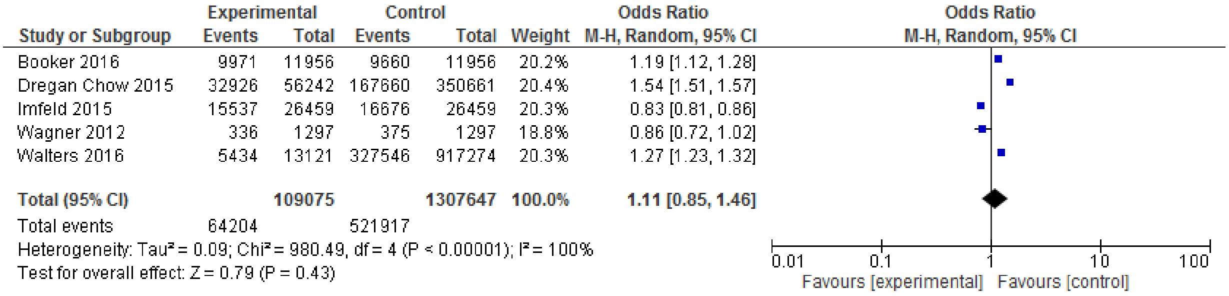

Supplement: S2 Fig — (PDF) [file pone.0194735.s002.pdf]
