# Supplementary material for: Predicting dementia from primary care records: A systematic review and meta-analysis
Source: PLoS One. 2018 Mar 29;13(3):e0194735. doi: 10.1371/journal.pone.0194735 (PMC5875793; doi:10.1371/journal.pone.0194735)

Figure 1: Forest Plot of comparison: Obesity, outcome: Dementia

	Cases		Controls			Odds Ratio
**Study or Subgroup**	**Events**	**Total**	**Events**	**Total**	**Weight**	**M-H, Random, 95% CI**
Booker 2016	717	11956	717	11956	44.7%	1.00 [0.90, 1.11]
Dregan Chow 2015	6224	56242	33561	350661	55.3%	1.18 [1.14, 1.21]
Imfeld 2015 benzos	1003	11524	1520	11524	0.0%	0.63 [0.58, 0.68]
Imfeld 2016 influenza	5	59	55	221	0.0%	0.28 [0.11, 0.73]
Imfeld Pernus 2013	3169	26459	4132	26459	0.0%	0.74 [0.70, 0.77]
Jick 2000	32	284	214	1080	0.0%	0.51 [0.35, 0.76]
Seshadri 2001	1184	19463	3157	19463	0.0%	0.33 [0.31, 0.36]
**Total (95% CI)**		**68198**		**362617**	**100.0%**	**1.09 [0.93, 1.28]**
Total events	6941		34278			

Heterogeneity: Tau² = 0.01; Chi² = 8.24, df = 1 (P = 0.004); I² = 88%
Test for overall effect: Z = 1.11 (P = 0.27)

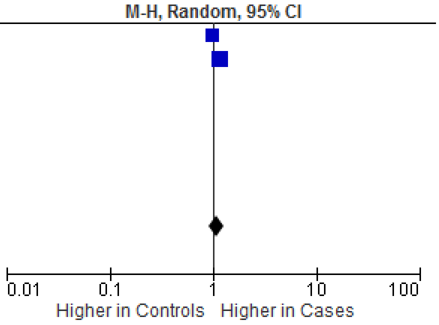

Figure 2: Forest Plot of comparison: Smoker, outcome: Dementia

	Cases		Control			Odds Ratio
**Study or Subgroup**	**Events**	**Total**	**Events**	**Total**	**Weight**	**M-H, Random, 95% CI**
Dregan Chow 2015	6314	56242	33181	350661	50.1%	1.21 [1.18, 1.25]
Dunn 2005 Lithium	41	284	85	1080	0.0%	1.98 [1.33, 2.94]
Imfeld 2015 benzos	2412	26459	2374	26459	0.0%	1.02 [0.96, 1.08]
Imfeld 2016 influenza	1518	19463	1652	19463	0.0%	0.91 [0.85, 0.98]
Imfeld Pernus 2013	1119	11524	1051	11524	0.0%	1.07 [0.98, 1.17]
Jick 2000	710	9954	395	9374	0.0%	1.75 [1.54, 1.98]
Seshadri 2001	10	59	32	221	0.0%	1.21 [0.55, 2.62]
Walters 2016	1468	12172	154468	857334	49.9%	0.62 [0.59, 0.66]
**Total (95% CI)**		**68414**		**1207995**	**100.0%**	**0.87 [0.45, 1.67]**
Total events	7782		187649			

Heterogeneity: Tau² = 0.22; Chi² = 445.21, df = 1 (P < 0.00001); I² = 100%
Test for overall effect: Z = 0.42 (P = 0.67)

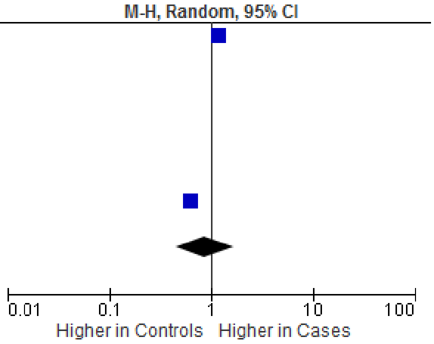

Figure 3: Forest plot of comparison: Depression, outcome: Dementia

	Cases		Control			Odds Ratio
**Study or Subgroup**	**Events**	**Total**	**Events**	**Total**	**Weight**	**M-H, Random, 95% CI**
Buntinx 1996	8	137	481	18966	4.7%	2.38 [1.16, 4.90]
Burton 2013	41	400	67	1353	11.3%	2.19 [1.46, 3.29]
Dregan Chow 2015	14492	56242	61804	350661	33.3%	1.62 [1.59, 1.66]
Imfeld 2015 benzos	4668	26459	3491	26459	0.0%	1.41 [1.34, 1.48]
Imfeld 2016 influenza	3995	19463	2796	19463	0.0%	1.54 [1.46, 1.62]
Imfeld Pernus 2013	2648	11524	1716	11524	0.0%	1.71 [1.59, 1.82]
Koehler 2015	58	1680	950	34111	18.0%	1.25 [0.95, 1.63]
Seshadri 2001	0	0	0	0		Not estimable
Walters 2016	2536	13121	98129	917274	32.7%	2.00 [1.91, 2.09]
**Total (95% CI)**		**71580**		**1322365**	**100.0%**	**1.75 [1.48, 2.07]**
Total events	17135		161431			

Heterogeneity: Tau² = 0.02; Chi² = 78.93, df = 4 (P < 0.00001); I² = 95%
Test for overall effect: Z = 6.50 (P < 0.00001)

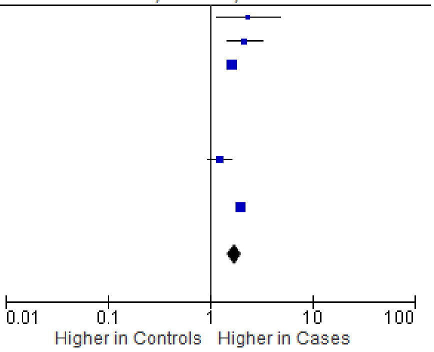

Figure 4: Forest plot of comparison: Coronary Heart Disease, outcome: Dementia

	Cases		Control			Odds Ratio
**Study or Subgroup**	**Events**	**Total**	**Events**	**Total**	**Weight**	**M-H, Random, 95% CI**
Booker 2016	3575	11956	3288	11956	24.4%	1.12 [1.06, 1.19]
Burton 2013	68	400	249	1353	18.1%	0.91 [0.68, 1.22]
Davies 2011	3106	9197	12938	39166	0.0%	1.03 [0.99, 1.08]
Dregan Chow 2015	12087	56242	73387	350661	24.7%	1.03 [1.01, 1.06]
Imfeld Pernus 2013	2484	11524	2729	11524	0.0%	0.89 [0.83, 0.94]
Jick 2000	61	284	320	1080	0.0%	0.65 [0.48, 0.89]
Ramakers 2007	16	74	30	125	8.3%	0.87 [0.44, 1.74]
Seshadri 2001	6	59	20	221	0.0%	1.14 [0.44, 2.97]
Walters 2016	2545	13121	119053	917274	24.5%	1.61 [1.54, 1.69]
**Total (95% CI)**		**81793**		**1281369**	**100.0%**	**1.13 [0.89, 1.45]**
Total events	18291		196007			

Heterogeneity: Tau² = 0.06; Chi² = 322.56, df = 4 (P < 0.00001); I² = 99%
Test for overall effect: Z = 1.02 (P = 0.31)

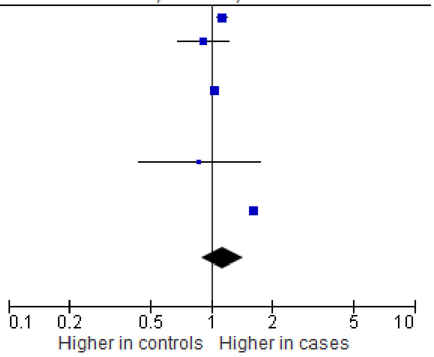

Figure 5: Forest plot of comparison: Hypertension, outcome: Dementia

Study or Subgroup	Cases Events	Cases Total	Control Events	Control Total	Weight	Odds Ratio M-H, Random, 95% CI
Booker 2016	7999	11956	7783	11956	30.6%	1.08 [1.03, 1.14]
Burton 2013	174	400	720	1353	14.6%	0.68 [0.54, 0.85]
Dregan Chow 2015	22794	56242	141606	350661	32.5%	1.01 [0.99, 1.02]
Imfeld 2015 benzos	12653	26459	13427	26459	0.0%	0.89 [0.86, 0.92]
Imfeld 2016 influenza	8761	19463	10358	19463	0.0%	0.72 [0.69, 0.75]
Imfeld Bodmer 2012	4926	11524	5424	11524	0.0%	0.84 [0.80, 0.88]
Jick 2000	80	284	385	1080	0.0%	0.71 [0.53, 0.94]
Ramakers 2007	31	74	45	125	3.5%	1.28 [0.71, 2.31]
Seshadri 2001	14	59	47	221	0.0%	1.15 [0.58, 2.28]
Wagner 2012	984	1297	895	1297	18.9%	1.41 [1.19, 1.68]
**Total (95% CI)**		**69969**		**365392**	**100.0%**	**1.04 [0.93, 1.17]**
Total events	31982		151049			

Heterogeneity: Tau² = 0.01; Chi² = 33.73, df = 4 (P < 0.00001); I² = 88%
Test for overall effect: Z = 0.73 (P = 0.47)

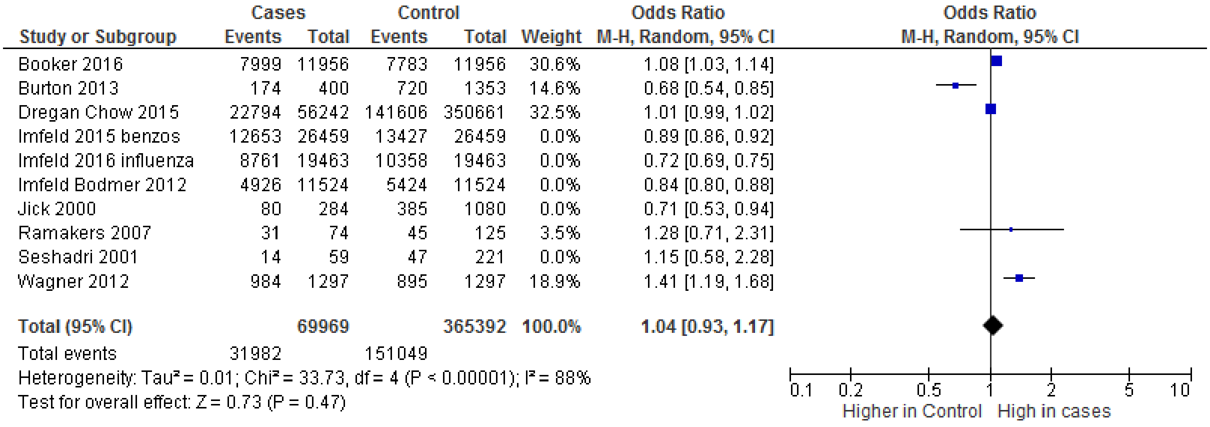

Figure 6: Forest plot of comparison: Dyslipidemia, outcome: Dementia

Study or Subgroup	Cases Events	Cases Total	Control Events	Control Total	Weight	Odds Ratio M-H, Random, 95% CI
Booker 2016	4304	11956	3993	11956	38.1%	1.12 [1.06, 1.18]
Burton 2013	43	400	200	1353	5.2%	0.69 [0.49, 0.99]
Imfeld 2015 benzos	4138	26459	3965	26459	39.3%	1.05 [1.00, 1.10]
Imfeld 2016 influenza	2998	19463	3096	19463	0.0%	0.96 [0.91, 1.02]
Imfeld Pernus 2013	1096	11524	1145	11524	0.0%	0.95 [0.87, 1.04]
Jick 2000	29	284	142	1080	0.0%	0.75 [0.49, 1.15]
Seshadri 2001	3	29	7	221	0.0%	3.53 [0.86, 14.48]
Wagner 2012	525	1297	475	1297	17.4%	1.18 [1.00, 1.38]
**Total (95% CI)**		**40112**		**41065**	**100.0%**	**1.08 [0.99, 1.17]**
Total events	9010		8633			

Heterogeneity: Tau² = 0.00; Chi² = 10.38, df = 3 (P = 0.02); I² = 71%
Test for overall effect: Z = 1.69 (P = 0.09)

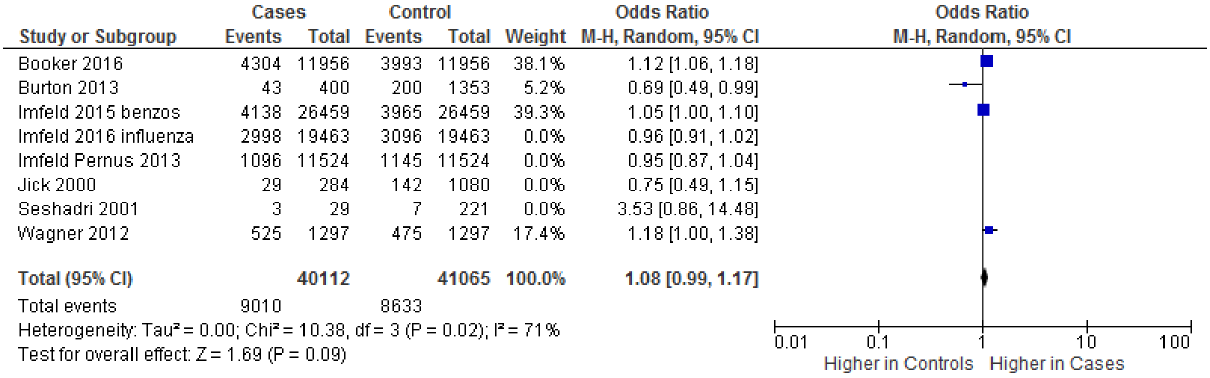

Figure 7: Forest plot of comparison: Atrial Fibrillation, outcome: Dementia

Study or Subgroup	Cases Events	Cases Total	Control Events	Control Total	Weight	Odds Ratio M-H, Random, 95% CI
Imfeld 2015 benzos	2413	26459	2397	26459	50.0%	1.01 [0.95, 1.07]
Imfeld 2016 influenza	1615	19463	2157	19463	0.0%	0.73 [0.68, 0.78]
Imfeld Pernus 2013	1331	11524	1244	11524	0.0%	1.08 [0.99, 1.17]
Walters 2016	1241	13121	38040	917274	50.0%	2.41 [2.28, 2.56]
**Total (95% CI)**		**39580**		**943733**	**100.0%**	**1.56 [0.65, 3.72]**
Total events	3654		40437			

Heterogeneity: Tau² = 0.39; Chi² = 430.43, df = 1 (P < 0.00001); I² = 100%
Test for overall effect: Z = 1.00 (P = 0.32)

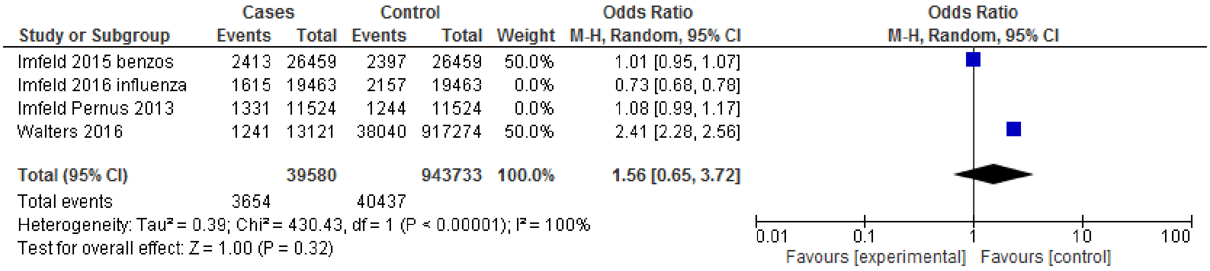

Figure 8: Forest plot of comparison: Heart Failure, outcome: Dementia

Study or Subgroup	cases Events	cases Total	Control Events	Control Total	Weight	Odds Ratio M-H, Random, 95% CI
Imfeld 2015 benzos	1412	26459	1602	26459	52.4%	0.87 [0.81, 0.94]
Imfeld 2016 influenza	947	19463	1478	19463	0.0%	0.62 [0.57, 0.68]
Imfeld Pernus 2013	1048	11524	1142	11524	0.0%	0.91 [0.83, 0.99]
Wagner 2012	525	1297	465	1297	47.6%	1.22 [1.04, 1.43]
**Total (95% CI)**		**27756**		**27756**	**100.0%**	**1.02 [0.74, 1.41]**
Total events	1937		2067			

Heterogeneity: Tau² = 0.05; Chi² = 13.68, df = 1 (P = 0.0002); I² = 93%
Test for overall effect: Z = 0.14 (P = 0.89)

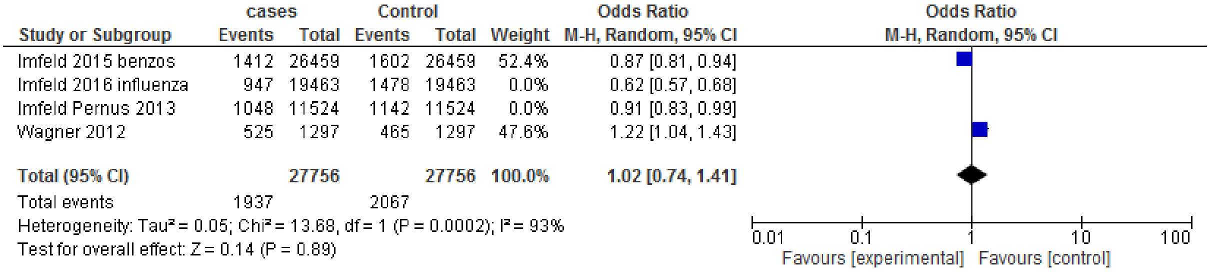

Figure 9: Forest plot of comparison: Diabetes, outcome: Dementia

Study or Subgroup	Cases Events	Cases Total	Control Events	Control Total	Weight	Odds Ratio M-H, Random, 95% CI
Booker 2016	4053	11956	3539	11956	26.8%	1.22 [1.15, 1.29]
Burton 2013	51	400	162	1353	4.3%	1.07 [0.77, 1.50]
Davies 2011	1363	9197	5629	39166	0.0%	1.04 [0.97, 1.11]
Dregan Chow 2015	8054	56242	43925	350661	30.2%	1.17 [1.14, 1.20]
Dunn 2005 Infections	772	9954	536	9374	0.0%	1.39 [1.24, 1.55]
Imfeld 2015 benzos	3084	26459	2979	26459	0.0%	1.04 [0.99, 1.10]
Imfeld 2016 influenza	2055	19463	2422	19463	0.0%	0.83 [0.78, 0.88]
Imfeld Bodmer 2012	1225	11524	1221	11524	0.0%	1.00 [0.92, 1.09]
Jick 2000	30	284	103	1080	0.0%	1.12 [0.73, 1.72]
Seshadri 2001	1	59	6	221	0.0%	0.62 [0.07, 5.23]
Wagner 2012	470	1297	322	1297	12.0%	1.72 [1.45, 2.04]
Walters 2016	1415	13121	81724	917274	26.7%	1.24 [1.17, 1.31]
**Total (95% CI)**		**83016**		**1282541**	**100.0%**	**1.25 [1.16, 1.35]**
Total events	14043		129672			

Heterogeneity: Tau² = 0.00; Chi² = 23.32, df = 4 (P = 0.0001); I² = 83%
Test for overall effect: Z = 5.90 (P < 0.00001)

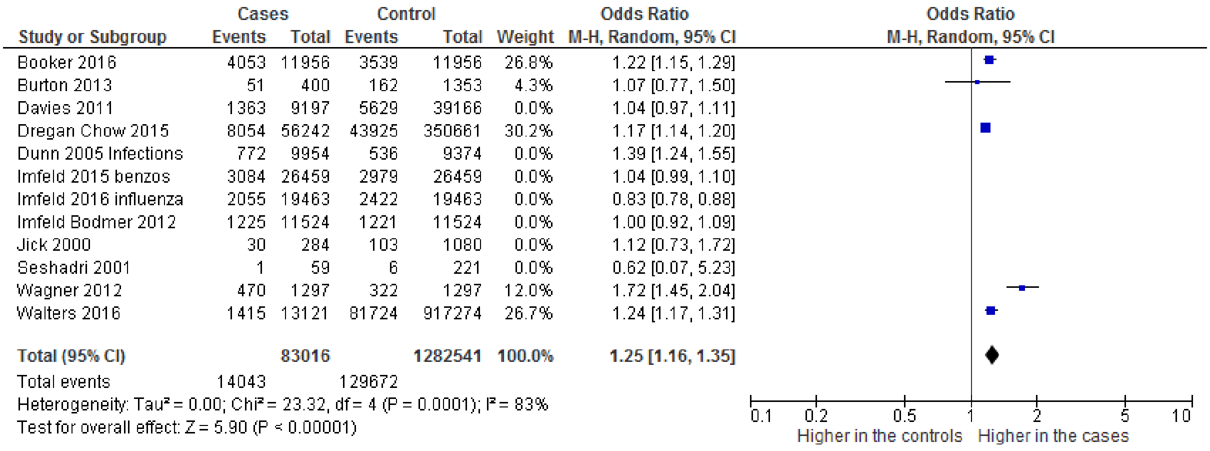

Figure 10: Forest plot of comparison: COPD, outcome: Dementia

Study or Subgroup	Cases Events	Cases Total	Control Events	Control Total	Weight	Odds Ratio M-H, Random, 95% CI
Dregan Chow 2015	5136	31083	65804	350661	100.0%	0.86 [0.83, 0.88]
Imfeld 2016 influenza	1050	19463	1351	19463	0.0%	0.76 [0.70, 0.83]
Imfeld Pernus 2013	333	7086	505	7086	0.0%	0.64 [0.56, 0.74]
**Total (95% CI)**		**31083**		**350661**	**100.0%**	**0.86 [0.83, 0.88]**
Total events	5136		65804			

Heterogeneity: Not applicable
Test for overall effect: Z = 9.73 (P < 0.00001)

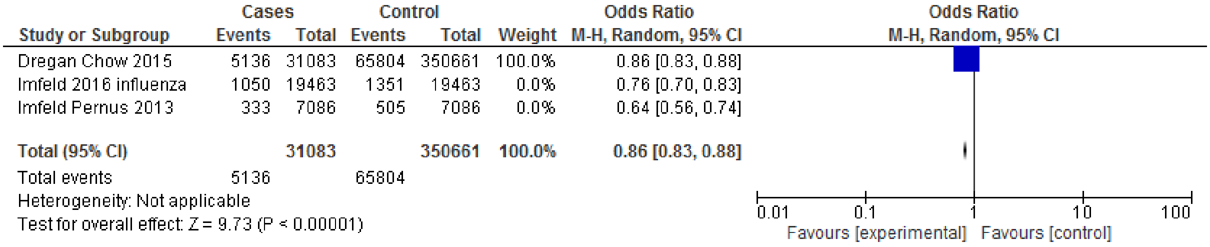

Figure 11: Forest plot of comparison: Inflammatory Conditions, outcome: Dementia

Study or Subgroup	Cases Events	Cases Total	Control Events	Control Total	Weight	Odds Ratio M-H, Fixed, 95% CI
Dregan Chow 2015	4096	56242	25581	350661	100.0%	1.00 [0.96, 1.03]
Dregan, Armstrong 2015	1378	4183	152172	458210	0.0%	0.99 [0.93, 1.05]
Imfeld 2016 influenza	215	19463	219	19463	0.0%	0.98 [0.81, 1.19]
Imfeld Pernus 2013	124	11524	122	11524	0.0%	1.02 [0.79, 1.31]
**Total (95% CI)**		**56242**		**350661**	**100.0%**	**1.00 [0.96, 1.03]**
Total events	4096		25581			

Heterogeneity: Not applicable
Test for overall effect: Z = 0.10 (P = 0.92)

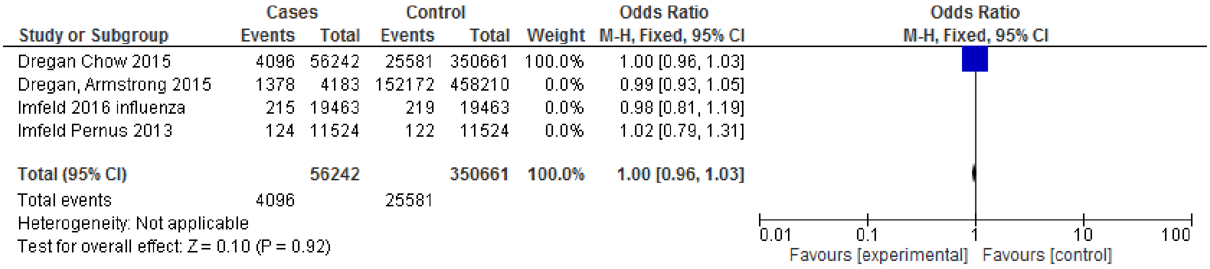

Figure 12: Forest plot of comparison: Statins, outcome: Dementia

Study or Subgroup	Experimental Events	Experimental Total	Control Events	Control Total	Weight	Odds Ratio M-H, Random, 95% CI
Booker 2016	4340	11956	4065	11956	25.5%	1.11 [1.05, 1.17]
Dregan Chow 2015	17332	56242	79682	350661	25.7%	1.51 [1.49, 1.54]
Imfeld 2015	7451	26459	7255	26459	0.0%	1.04 [1.00, 1.08]
Imfeld Pernus 2013	2441	11524	2451	11524	0.0%	0.99 [0.93, 1.06]
Jick 2000	12	284	100	1080	0.0%	0.43 [0.23, 0.80]
Wagner 2012	343	1297	324	1297	23.3%	1.08 [0.91, 1.29]
Walters 2016	2272	13121	165549	917274	25.6%	0.95 [0.91, 1.00]
**Total (95% CI)**		**82616**		**1281188**	**100.0%**	**1.15 [0.87, 1.51]**
Total events	24287		249620			

Heterogeneity: Tau² = 0.08; Chi² = 418.19, df = 3 (P < 0.00001); I² = 99%
Test for overall effect: Z = 0.98 (P = 0.33)

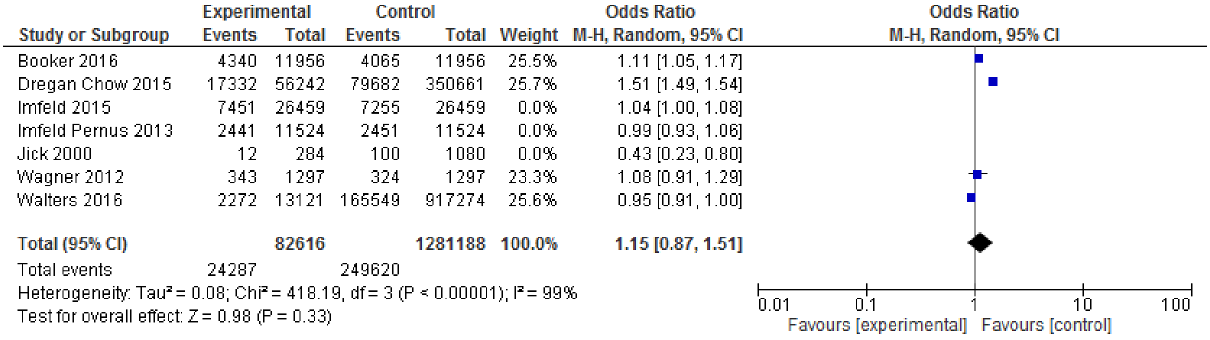

Supplement: S3 Fig — (PDF) [file pone.0194735.s003.pdf]
