# Supplementary material for: Predicting dementia from primary care records: A systematic review and meta-analysis
Source: PLoS One. 2018 Mar 29;13(3):e0194735. doi: 10.1371/journal.pone.0194735 (PMC5875793; doi:10.1371/journal.pone.0194735)
Supplement: S1 Table — (PDF) [file pone.0194735.s004.pdf]

Study	Ottowa Newcastle Scale									RoBANS					
Case-control Studies	Selection				Comparability	Exposure			Total N Stars						
	1) case definition validation	2) Representative-ness	3) selection of controls	4) definition of controls	1) Comparability of cases and controls	1) Ascertainment of Exposure	2) Same method of ascertainment for cases and controls	3) Non-response rate		Selection of participants	Confounding Variables	Measurement of exposure	of outcome assessments	Incmplete outcome data	Selective outcome reporting
Booker 2016		*	*	*	**	*	*	*	8	low risk	low risk	low risk	low risk	low risk	low risk
Burton 2012		*	*	*	**	*	*	*	8	low risk	low risk	low risk	low risk	low risk	low risk
Davies 2011	*	*	*		**	*	*	*	8	low risk	low risk	low risk	low risk	low risk	low risk
Dregan 2015 anti-inflam drugs		*	*	*	**	*	*	*	8	low risk	low risk	low risk	low risk	low risk	low risk
Dunn 2005 Lithium	*	*	*	*	*	*	*	*	8	low risk	unclear ris	low risk	low risk	low risk	low risk
Dunn 2005 Infectious I	*	*	*	*	**	*	*	*	9	low risk	low risk	low risk	low risk	low risk	low risk
Imfeld 2012	*	*	*	*	**	*	*	*	9	low risk	low risk	low risk	low risk	low risk	low risk
Imfeld 2013 Seizures	*	*	*	*	**	*	*	*	9	low risk	low risk	low risk	low risk	low risk	low risk
Imfeld 2013 Epidemiol	*	*	*	*	**	*	*	*	9	low risk	low risk	low risk	low risk	low risk	low risk
Imfeld 2015	*	*	*	*	**	*	*	*	9	low risk	low risk	low risk	low risk	low risk	low risk
Imfeld 2016	*	*	*	*	**	*	*	*	9	low risk	low risk	low risk	low risk	low risk	low risk
Jick 2000	*		*	*	**	*	*	*	8	unclear	low risk	low risk	low risk	low risk	low risk
Ramakers 2007		*	*	*	**	*	*	*	8	low risk	low risk	low risk	low risk	low risk	low risk
Seshadri 2001	*	*	*		**	*	*	*	8	low risk	low risk	low risk	low risk	low risk	low risk
Wagner 2012		*	*	*	**	*	*	*	8	low risk	low risk	low risk	low risk	low risk	low risk
Cohort studies	Selection				Comparability	Outcome			Total						
	1) Representativeness of the exposed cohort	Selection of non-exposed cohort	ascertainment of exposure	outcome not present at start	Comparability of cohorts on basis of design or analysis	Assessment of outcome	Follow up long enough >10 years	Adequacy of follow up							
Buntinx 1996	*	*	*	*	**	*		*	8	low risk	low risk	low risk	low risk	low risk	low risk
Dregan 2015 inflamma	*	*	*	*	**	*		*	8	low risk	low risk	low risk	low risk	low risk	low risk
Kessing 2008	*	*	*	*	**	*			7	low risk	low risk	low risk	low risk	low risk	low risk
Koehler 2015	*	*	*	*	**	*	*	*	9	low risk	low risk	low risk	low risk	low risk	low risk
Walters 2016	*	*	*	*	*	*		*	8	low risk	unclear ris	low risk	low risk	low risk	low risk
